# Quantitative Macromolecular Proton Fraction Mapping Reveals Altered Cortical Myelin Profile in Schizophrenia Spectrum Disorders

**DOI:** 10.1093/texcom/tgab015

**Published:** 2021-02-24

**Authors:** Yu Veronica Sui, Hilary Bertisch, Hong-Hsi Lee, Pippa Storey, James S Babb, Donald C Goff, Alexey Samsonov, Mariana Lazar

**Affiliations:** Department of Radiology, NYU Grossman School of Medicine, New York, NY 10016, USA; Department of Rehabilitation Medicine, NYU Grossman School of Medicine, New York, NY 10016, USA; Department of Radiology, NYU Grossman School of Medicine, New York, NY 10016, USA; Department of Radiology, NYU Grossman School of Medicine, New York, NY 10016, USA; Department of Radiology, NYU Grossman School of Medicine, New York, NY 10016, USA; Department of Psychiatry, NYU Grossman School of Medicine, New York, NY 10016, USA; Nathan Kline Institute, Orangeburg, New York, NY 10016, USA; Department of Radiology, University of Wisconsin–Madison, Madison, WI 53705, USA; Department of Radiology, NYU Grossman School of Medicine, New York, NY 10016, USA

**Keywords:** cerebral cortex, magnetization transfer, myelin, schizophrenia, working memory

## Abstract

Myelin abnormalities have been reported in schizophrenia spectrum disorders (SSD) in white matter. However, *in vivo* examinations of cortical myeloarchitecture in SSD, especially those using quantitative measures, are limited. Here, we employed macromolecular proton fraction (MPF) obtained from quantitative magnetization transfer imaging to characterize intracortical myelin organization in 30 SSD patients versus 34 healthy control (HC) participants. We constructed cortical myelin profiles by extracting MPF values at various cortical depths and quantified their shape using a nonlinearity index (NLI). To delineate the association of illness duration with myelin changes, SSD patients were further divided into 3 duration groups. Between-group comparisons revealed reduced NLI in the SSD group with the longest illness duration (>5.5 years) compared with HC predominantly in bilateral prefrontal areas. Within the SSD group, cortical NLI decreased with disease duration and was positively associated with a measure of spatial working memory capacity as well as with cortical thickness (CT). Layer-specific analyses suggested that NLI decreases in the long-duration SSD group may arise in part from significantly increased MPF values in the midcortical layers. The current study reveals cortical myelin profile changes in SSD with illness progression, which may reflect an abnormal compensatory mechanism of the disorder.

## Introduction

Schizophrenia spectrum disorders (SSD), characterized by a range of symptoms including psychosis and cognitive dysfunction, are debilitating mental disorders that tend to have devastating consequences for patients and their families. Despite significant advances in the treatment, the etiology and mechanisms underlying SSD have remained elusive.

Among theories proposed to date, the dysmyelination hypothesis has gained attention in recent years along with the accumulation of histological evidence and advancements in *in vivo* myelin imaging techniques. Based on previous findings in patients with schizophrenia or schizoaffective disorder, including reduced oligodendrocytes density and oligodendrocyte-associated proteins (Flynn et al. 2003; Takahashi et al. 2011; Goudriaan et al. 2014), structural alterations in myelin lamellae (Uranova et al. 2011), and abnormalities in overall myelin content distribution (Palaniyappan et al. 2013; Palaniyappan et al. 2018; Wei et al. 2018), this hypothesis emphasizes the role of myelin and oligodendrocyte deficits in SSD pathophysiology and proposes that these deficits disrupt signal integration and network connectivity, which in turn impacts brain function.

A relatively large number of studies employing magnetic resonance imaging (MRI) have supported the dysmyelination hypothesis, particularly in white matter. In contrast, abnormalities in cortical myelin have been less investigated *in vivo*. Although the majority of myelin content resides in deep white matter tracts, there is also a substantial number of myelinated fibers within the cerebral cortex (Nieuwenhuys 2013; Redlich and Lim 2019). Importantly, postmortem studies in schizophrenia have observed more prominent myelin and oligodendrocyte deficits in the cortex than in white matter (Bartzokis 2012). Nevertheless, investigations of intracortical myelin changes in SSD using MRI remained limited until recently by a lack of adequate imaging methods and poor image resolution.

*In vivo* myelin mapping in the cerebral cortex requires metrics that are both sensitive and specific to myelin content despite the complex cortical architecture. Previous studies have employed metrics based on the magnetization transfer (MT) effect, such as the magnetization transfer ratio (MTR) (Henkelman et al. 2001; Palaniyappan et al. 2018; Wei et al. 2018), and relaxometry-based ones including longitudinal relaxation rate (R_1_) and myelin water fraction (Stuber et al. 2014; Does 2018; West et al. 2018). More recently, the ratio of T_1_- and T_2_-weighted image intensity (T_1_w/T_2_w) was proposed and used as a cortical myelin proxy in the Human Connectome Project (Glasser and Van Essen 2011; Glasser et al. 2013). Using MTR and T_1_w/T_2_w, decreased cortical myelin was found in patients with schizophrenia and schizoaffective disorder compared with controls (Iwatani et al. 2015; Wei et al. 2018). However, variation exists in terms of the extent, location, and direction of cortical myelin alterations across studies, likely confounded by the age and disease stage of patient groups involved and dependent on the cortical depth examined (Wei et al. 2020).

The macromolecular proton fraction (MPF) based on MT imaging and quantitative parameter fitting is one of the recently proposed metrics that show great promise as myelin measures. MPF reflects the molar fraction of protons bound with macromolecules, which in the brain are predominantly myelin lipids and large proteins. Compared with traditional MTR, which is considered a semiquantitative metric (Yarnykh 2012; Heath et al. 2018), MPF is fitted by employing additional relaxometry measurements in combination with specific models describing between-pool MT dynamics and is hence a quantitative measure. Another advantage of MPF over relaxometry-based methods is its robustness against the susceptibility effects caused by iron content in the brain (Yarnykh et al. 2018). Using animal models and postmortem brain tissue of MS patients, MPF has been validated as a more reliable myelin marker through extensive comparisons with histology (Samsonov et al. 2010; Underhill et al. 2011; Khodanovich et al. 2017; Khodanovich et al. 2019).

Using MPF, the present study aimed to characterize cortical myelin alterations in young adult SSD patients versus healthy control (HC) participants and their variation with disease progression. Mean region of interest (ROI) values, which reflect averaged signal intensity of the region, may be less sensitive in detecting fine changes when the tissue structure is highly inhomogeneous, such as in the cortex, where multiple distinct layers exist. Thus, to further characterize myelin alterations in patients with SSD, we generated cortical myelin profiles perpendicular to the cortical ribbon and calculated their nonlinearity index (NLI), as recently proposed by Sprooten et al. (2019) with T_1_ images. Cortical myelin NLI was suggested to be sensitive to subtle deviations from normal myelin organization caused by layer-specific changes and to closely relate to the regions designated functions (Sprooten et al. 2019). Further, given the important role of myelin in optimal neural communication, we tested associations of mean MPF and NLI with spatial working memory capacity, a key cognitive feature known to be affected in SSD. Finally, we hypothesized that changes in cortical myelination may also be associated with cortical thinning in SSD. Although reduced cortical thickness (CT) is one of the most robust findings in SSD, its origins and effects on microstructural cortical organization remain poorly understood (Schultz et al. 2010; Takayanagi et al. 2013; Kong et al. 2015).

## Materials and Methods

### Participants

Data included here were acquired as part of a larger study aiming to investigate dysmyelination deficits in psychotic spectrum disorders. Patients and comparison HC were recruited through advertising within community through online and onsite postings across NYU Hospitals and Clinics and local chapter of National Alliance on Mental Illness, online advertising through Craigslist and Research Match, and referrals from NYU Psychiatry Department and Bellevue Hospital Clinics and Programs. Inclusion criteria for the SSD group examined here were a diagnosis of schizophrenia or schizoaffective disorder and an age range of 18–31 years. Comparison HC participants within the same age range were also recruited. Exclusion criteria for HC were serious or unstable medical illness, history of psychiatric disorders, or first-degree relatives with a diagnosis of schizophrenia, schizoaffective, or bipolar disorder. Exclusion criteria for both groups included substance abuse within 6 months prior to study participation, presence of organic brain disorders, or brain trauma with loss of consciousness for longer than 30 min. After observation of inclusion and exclusion criteria and elimination of datasets presenting image artifacts (see Supplementary Material), 30 individuals in the SSD group and 34 in the HC group were retained for analyses (Table 1). All but 4 patients were currently taking medications, including first-generation antipsychotics (two patients), second-generation antipsychotics (21 patients), antidepressants (8 patients), anxiolytics (5 patients), mood stabilizers (5 patients) and anticholinergics (two patients). A list of current medications for each patient is included in the Supplementary Material (Supplementary Table 1). As reliable data on antipsychotic medication dosage and treatment duration were not available for many patients, findings were not adjusted for antipsychotic dose and cumulative antipsychotic exposure.

**Table 1:** Demographic and clinical characteristics

	HC	Patients with SSD			
		SSD	SSD-S	SSD-M	SSD-L
Number of subjects	34	30	11	10	9
Sex, F/M	15/19	10/20	3/8	2/8	5/4
Age in years					
Mean (SD)	25.0 (3.5)	25.2 (2.9)	24.1 (2.7)	24.5 (2.9)	27.2 (2.4)
[Range]	[18.0–31.3]	[20.8–30.7]	[20.8–28.5]	[21.2–29.9]	[23.7–30.7]
Symbol Span score					
Mean (SD)	28.1 (7.6)	20.1 (7.7)	23.1 (7.8)	18.5 (5.3)	18.0 (9.5)
[Range]	[9–40]	[10–38]	[10–32]	[10–25]	[10–38]
Diagnosis					
Schizophrenia/Schizoaffective	—	15/15	6/5	5/5	4/5
Illness duration					
Mean (SD)	—	4.8 (4.0)	1.5 (0.6)	4.0 (0.8)	10.3 (2.8)
[Range]	—	[0.7–13.0]	[0.7–2.4]	[2.5–5.2]	[5.9–13.0]
SAPS					
Mean (SD)	—	25.5 (16.8)	16.1 (10.5)	31.7 (23.7)	29 (7.7)
[range]	—	[0–76]	[0–33]	[0–76]	[19–41]
SANS					
Mean (SD)	—	15.7 (10.2)	16.2 (12.9)	14.1 (9.6)	16.9 (8.3)
[Range]	—	[4–48]	[5–48]	[4–33]	[6–28]
Medication					
Antipsychotics (N)	—	23	7	9	7
Antidepressants (N)	—	8	3	3	2

All participants provided written consent to participate according to the requirements of the Institutional Review Board at New York University Grossman School of Medicine.

### Clinical and Cognitive Assessments

The diagnostic interview for genetic studies (DIGS) was administered to all participants to confirm an SSD diagnosis in patients and the lack of a psychiatric diagnosis in HC. The Scale for the Assessment of Negative Symptoms (SANS) and the Scale for the Assessment of Positive Symptoms (SAPS) were used to assess patients’ clinical symptoms (Table 1). All patients were further divided into 3 duration groups based on reported illness duration in the DIGS: short duration 0–2.5 years (SSD-S), medium duration 2.5–5.5 years (SSD-M), and long duration 5.5+ years (SSD-L). Illness duration was defined by the time in years between current age and the age of psychotic symptom onset. In our preliminary analyses, we also explored employing two SSD duration groups (illness duration shorter/longer than 5 years), which resulted in similar trends in the observed myelin changes as for the 3 groups (Sui et al. 2020). The grouping scheme used here was chosen since it appeared to best reflect the effect of illness duration on cortical myelin changes.

To test the association of quantitative MRI metrics with cognitive function, the Symbol Span test from the Wechsler Memory Scale fourth edition, which provides a measure of spatial working memory capacity, was administered as part of a larger cognitive test battery including selective tests from the MATRICS Consensus Cognitive Battery (Wechsler 1945; Nuechterlein and Green 2006). Symbol Span raw score was used in the analysis to preserve natural variations among subjects. One patient in the SSD-L group could not provide an exact age for psychosis onset and had a missing Symbol Span score. This patient was hence excluded from the correlation analyses relating myelin metrics to Symbol Span score and illness duration.

### MRI Acquisition

Participants were scanned on a Siemens 3T Prisma scanner using a 64-channel head coil. A 20-channel head coil was used for one patient who did not fit in the 64-channel coil. A fast MPF mapping protocol (Yarnykh 2016) based on a 3-dimensional gradient-echo MT-weighted acquisition (repetition time (TR) = 29 ms, echo time (TE) = 2.43 ms, flip angle (FA) = 10°) and two non-MT-weighted variable-flip-angle (VFA) spoiled gradient-echo acquisitions (TR = 21 ms, TE = 2.43 ms, FA = 4° and 25°), was implemented. All gradient-echo volumes were acquired using an isotropic voxel size of 1.5 mm with Cartesian sampling. In the MT-weighted acquisition, off resonance saturation was achieved by applying a Gaussian pulse with FA = 560°, pulse duration = 12.3 ms, and offset frequency = 4 kHz. For field inhomogeneity correction, the Siemens turboFLASH B_1_ mapping sequence was used to obtain B_1_+ field maps with a voxel size of 2.67 × 2.67 × 5.8 mm^3^. B_0_ field maps were estimated using FSL topup and a set of 10 b = 0 s/mm^2^ diffusion images acquired with two opposite polarities in the phase encoding direction (anterior to posterior and posterior to anterior), which had identical voxel size to the MPF mapping protocol.

For anatomical reference, high resolution (0.8 mm isotropic voxels) 3-dimensional T_1_w magnetization-prepared rapid gradient-echo images were acquired using the Human Connectome Lifetime protocol (Glasser et al. 2013). Imaging parameters included TR = 2400 ms, TE = 2.24 ms, TI = 1060 ms, and FA = 8°.

### MRI Processing

FreeSurfer, FSL, and Matlab were used for image processing, statistical analysis, and graphing. MT-weighted and VFA images were first processed to mitigate Gibbs ringing artifacts (Kellner et al. 2016), brain-extracted using bet utility in FSL (Smith 2002), and coregistered using FSL flirt (Jenkinson et al. 2002). All images remained in subject space throughout the procedure. For field inhomogeneity correction, B_0_ and B_1_ field maps were coregistered to the MT space and brain-masked. All processing steps were visually checked for quality assurance. Quantitative MPF parametric maps were generated using in-house-developed scripts based on a previously described method of modified cross-relaxation imaging (Yarnykh 2012; Mossahebi et al. 2014; Samsonov et al. 2014).

Cortical parcellation and surface reconstruction were conducted using FreeSurfer recon-all on individual T_1_w images. The Desikan–Killiany atlas with 34 parcels in each hemisphere was used in the analysis. Because of head placement in the imaging field of view, slight variations in image intensity were present at the edge of the imaging slab which resulted in poor MPF fitting for some subjects in limited areas in lower temporal lobe (i.e., inferior temporal, temporal pole, fusiform, entorhinal, and parahippocampal). The affected voxels were removed, leading to missing values for 5 subjects in selective regions in temporal lobe.

### Myelin Profile NLI

**Figure 1:**
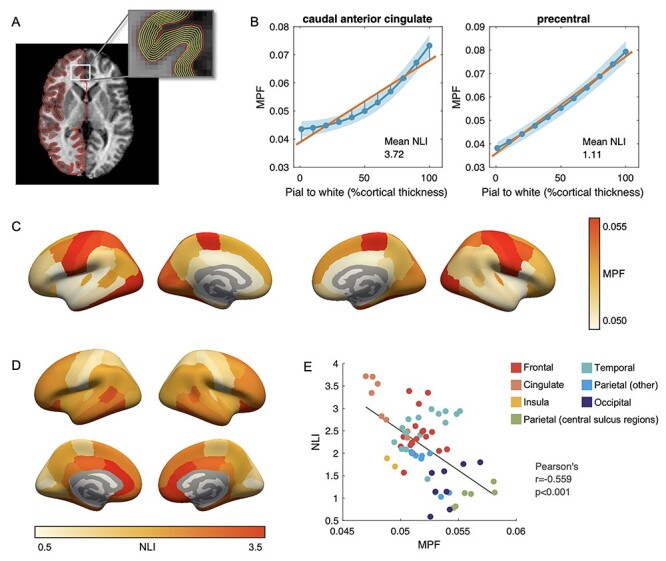
(*A*) Depths sampled within cortical ribbon. (*B*) Average cortical MPF profiles and mean NLI in the HC group for two representative regions: caudal anterior cingulate (high NLI) and precentral (low NLI) regions from the left hemisphere. Solid blue lines depict mean MPF values on each sampled surface, whereas the shaded areas mark their one SD range. (*C*) Mean MPF across cortical regions in the HC group. (*D*) Mean NLI across cortical regions in the HC group. (*E*) Correlation between MPF and NLI across 68 bilateral cortical regions in the HC group. Color indicates the lobe/area the region is located.

For each subject, the brain-only MPF map was registered to the T_1_w image using FreeSurfer’s bbregister. Nine uniformly spaced layers were expanded within the cortical ribbon between the white and pial surface, each with 10% cortical thickness apart (Fig. 1*A*). MPF values were extracted from the 9 expanded surfaces and from near the pial (1% CT) and white (99% CT) surfaces using FreeSurfer’s mri_vol2surf. These 11 datapoints were then used to construct a cortical myelin profile (Fig. 1*B*). The 1% and 99% cortical depths were chosen in order to mitigate contamination from CSF or white matter at the cortical ribbon boundaries. To quantify the profile shape of each region, a cortical myelin NLI was calculated as the root-mean-square deviation of the MPF profile curve across layers from a linear fitting line,$\hat{y}(x)=a+ bx,$ of MPF (*y*) over cortical depths *x* = [1,10,20,30,40,50,60,70,80,90,99]:

(1) \begin{equation*} \mathrm{NLI}=\sqrt{\frac{\sum_{i=1}^{11}{\left({y}_i-{\hat{y}}_i\right)}^2}{11}} \end{equation*}

For easier discussion, NLI values across all datasets and regions were standardized to a mean of 2 and a standard deviation (SD) of 1:

(2) \begin{equation*} \mathrm{NLI}=\frac{\mathrm{NLI}-\mu}{\sigma}+2, \end{equation*}

where μ and σ are the mean and SD of NLI values across all regions and from all datasets.

To assess the validity of MPF cortical myelin profile, we employed a self-similarity-based super-resolution method that introduced anatomical details of the high-resolution T_1_w images to the MT-weighted and VFA images and artificially increased their resolution by a factor of 2 (Manjon et al. 2010; Lee et al. 2019). NLI obtained using these images showed good correspondence with the original NLI in the HC group (Supplementary Fig. 1). Detailed description of these steps is included in the Supplementary Material.

### Statistical Analyses

Between-group comparisons of MRI metrics were performed using two-tailed independent samples *t*-tests. Pearson’s correlation was used to examine the association between MRI metrics and patients’ illness duration. Mediation analysis was conducted to assess the mediation effect of midcortical MPF values on NLI changes. To account for the potential effect of age and sex, we repeated group comparisons and correlation adjusting for age and sex using analysis of covariance and partial correlation respectively. Relationships between MRI metrics and Symbol Span raw score were tested using partial correlation controlling for subject’s age, sex, and illness duration. The Bonferroni correction was used to adjust for multiple comparisons, with the significance level set at Bonferroni corrected *P* < 0.05. Certain test results that were only significant without the Bonferroni correction (P^*^ < 0.05, where P^*^ is the uncorrected *P* value) are also reported as trends in observation of limited statistical power because of sample size. The correlation between MPF and NLI in the HC (3.1) and SSD (3.4) groups was conducted across cortical regions and thus did not necessitate correcting for multiple comparisons.

## Results

### Myelin Profiles Across Healthy Brain

Regional variations across the cortex were observed for MPF and NLI in the HC group (Fig. 1*C*,*D*). Consistent with well-defined cortical myelin distribution, higher MPF values were found in regions along the central sulcus and in the occipital area. An inverse relationship between MPF and NLI group means was found across regions (Pearson’s *r* = −0.559, *P* < 0.001; Fig. 1*E*), where highly myelinated regions showed lower profile NLI while regions across the prefrontal, cingulate, and temporal lobe areas with relative lighter myelination showed higher NLI values.

### MPF and CT Changes with Illness Duration

**Figure 2:**
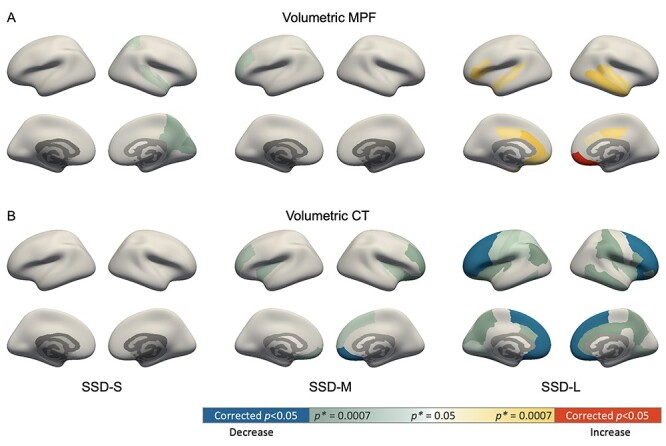
Mean MPF (*A*) and CT (*B*) changes in 3 SSD duration groups. Color indicates differences in patient groups compared with HC: blue and red denotes significant lower and higher values in the SSD groups than HC, respectively; green and yellow gradient denotes trend level differences with the color gradient indicating the magnitude of uncorrected *P* value (P^*^) according to the color bar.

ROI analysis showed significantly increased mean MPF for the SSD-L group compared with HC in the right medial orbitofrontal region (Bonferroni corrected *P* = 0.006), and increases at a trend level across extended areas (P^*^ < 0.05; Fig. 2*A*). Conversely, MPF was decreased at a trend level in the SSD-S group compared with HC in multiple ROIs (P^*^ < 0.05; for full statistics, see Supplementary Table 2).

Differences in CT between the HC and SSD duration groups (Fig. 2*B*; Supplementary Table 3) were also examined for comparison with existing literature. No significant or trend level differences were noted between SSD-S and HC, whereas for SSD-M, significantly reduced CT was observed in right medial orbitofrontal region (corrected *P* = 0.012). For the SSD-L group, significant CT decreases were found in extended areas primarily in the frontal region (corrected *P* < 0.05).

### NLI Changes with Illness Duration

**Figure 3:**
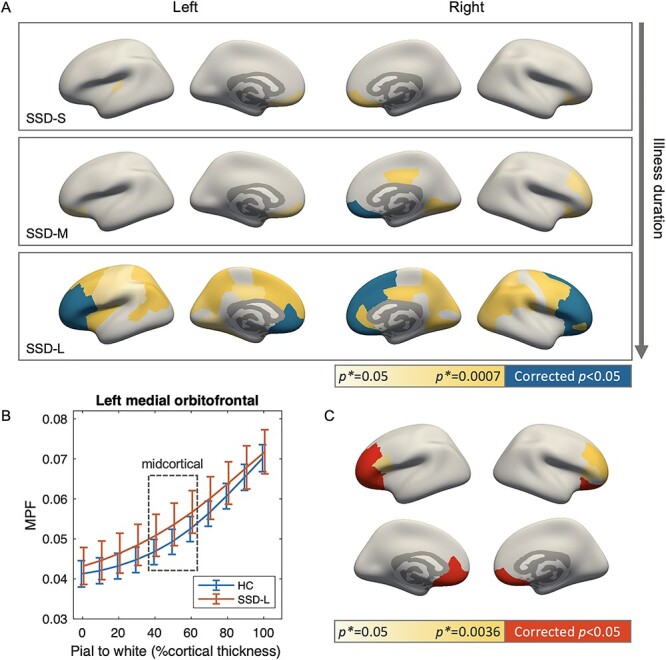
NLI and midcortical MPF changes in patients with SSD compared with the HC group. (*A*) Reduced NLI in the SSD groups, with panels from top to bottom showing results for short, medium, and long illness duration groups, respectively. Color of the regions indicates group comparison *P* values: blue denotes significant decreased NLI in the SSD groups compared with HC after the Bonferroni correction; yellow denotes trend level differences with color gradient indicating P^*^ magnitude according to the color bar. (*B*) Cortical myelin profile of the HC and SSD-L group from left hemisphere medial orbitofrontal region, showing a representative pattern of increased MPF in midcortical section for SSD-L. Error bars mark one SD range. Datapoints for subject groups are slightly shifted horizontally to prevent overlapping. (*C*) Regions with increased MPF values in midcortical layers in SSD-L compared with HC, examined post hoc among regions with significant NLI differences as shown in (*A*) bottom panel. Color indicates comparison *P* values: red denotes significant midcortical MPF increases in SSD-L compared with HC after the Bonferroni correction; yellow denotes trend level differences with color gradient indicating the magnitude of P^*^.

Significantly decreased NLI (corrected *P* < 0.05) were found in SSD-L compared with the HC group across bilateral prefrontal and anterior cingulate areas (Fig. 3*A*). For the SSD-M group, significant NLI decrease was found in the right hemisphere medial orbitofrontal region (corrected *P* = 0.013). Only trend-level NLI differences were noted for the SSD-S group compared with HC (P^*^ < 0.05). These results were robust after controlling for subject’s age and sex, except for the differences between HC and SSD-L in left pars opercularis and right caudal anterior cingulate, which switched to trend level differences (for full statistics, see Supplementary Table 4).

Upon visual inspection, a pattern of increased MPF values at midcortical surfaces was observed in the SSD-L group compared with HC (Fig. 3*B*). We thus compared average MPF values in the midcortical section (40%–60% cortical thickness position) of regions with significant NLI differences between HC and SSD-L. Significantly increased MPF (corrected *P* < 0.05) in midcortical section was found in 8 out of the tested 14 regions, primarily across prefrontal areas (Fig. 3*C*; Supplementary Table 5). Further mediation analysis examining the natural indirect effect of midcortical MPF indicated that MPF in the midcortical layers mediated the group differences in NLI. Approximately 16%–35% of NLI differences between the HC and SSD-L group were explained by the mediation effect of midcortical MPF values for bilateral medial and lateral orbitofrontal regions and right hemisphere pars orbitalis (Supplementary Table 5). Apart from increased midcortical MPF, NLI changes in other regions were likely to arise from subtle changes along the entire profile, including increased MPF near the pial surface accompanied with decreased MPF near white matter boundary; however, these observations were not statistically significant.

**Figure 4:**
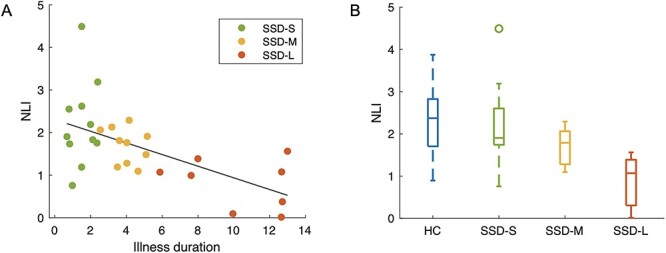
(*A*) Scatter plot of NLI values in left hemisphere pars orbitalis against illness duration for all SSD patients. The least squares regression line describing NLI as a function of illness duration within the SSD group is shown. (*B*) Box plot illustrates NLI value distributions in each group.

Within the 14 regions with significant between-group NLI differences, we further tested for association between NLI and illness duration using Pearson’s correlation. A significant negative association was observed for left pars orbitalis region (Pearson’s *r* = −0.59, corrected *P* = 0.010; Fig. 4*A*) with trend level associations noted in several other regions (P^*^ < 0.05; Supplementary Table 6). The negative correlation in left pars orbitalis remained significant after controlling for age and sex (Supplementary Table 6).

### Association of NLI with MPF and CT in SSD

**Figure 5:**
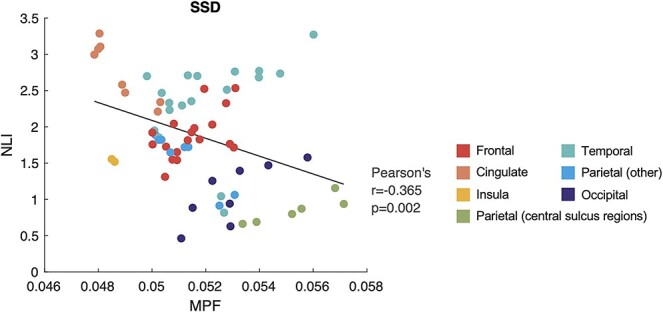
Correlation between mean MPF and NLI across 68 bilateral cortical regions in the SSD group. Color indicates the lobe/area each ROI is located.

In the SSD group, mean NLI was found to associate with mean MPF across regions (Fig. 5), which was consistent with the result in HC (Fig. 1*E*). Although the correlation coefficient in SSD (Pearson’s *r* = −0.365, *P* = 0.002) was slightly smaller than in HC (Pearson’s *r* = −0.559, *P* < 0.001), no significant group × MPF interaction in predicting NLI was noted by the regression analysis.

**Figure 6:**
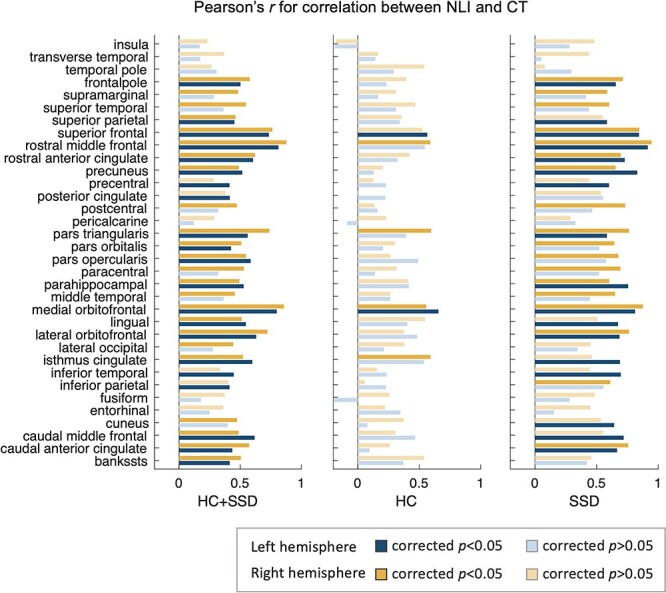
Pearson’s *r* of correlation between NLI and CT in bilateral brain areas. Significant associations are found in a large number of regions for all subjects (left column) and the patient group (right column), with only few significant associations noted in the HC group (middle column).

Within regions, significant positive correlations were found between NLI and CT in 45 out of the 68 bilateral ROIs (corrected *P* < 0.05; Fig. 6) for the HC and SSD participants combined. Within the SSD group, similar relationships were found in 35 regions across the brain (left hemisphere 17; right hemisphere 18), whereas for the HC group, only 6 regions showed significant positive correlation after the Bonferroni correction (Fig. 6). Therefore, positive correlation between NLI and CT across groups appeared to be contributed by both the relationship within the SSD group and the systematic variations between HC and SSD group. These findings were robust after adding subject’s age and sex as covariates in the correlation (Supplementary Fig. 2).

### NLI Correlates with Working Memory Capacity

**Figure 7:**
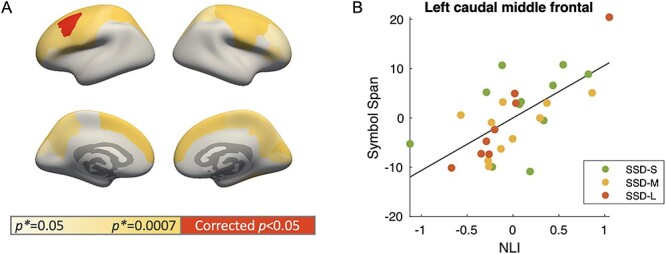
(*A*) Region-wise partial correlation between NLI and Symbol Span score within the SSD group controlling for age, sex, and illness duration. Color indicates the magnitude of *P* values of the partial correlation: red denotes significant partial correlation after the Bonferroni correction. (*B*) Partial plot of the significant positive correlation in left caudal middle frontal region.

Within the SSD group, NLI was found to be positively related to Symbol Span score controlling for the effect of subject’s age, sex, and illness duration (Fig. 7*A*). This relationship was observed in the left caudal middle frontal region at a significant level (Pearson’s *r* = 0.658, corrected *P* = 0.018; Fig. 7*B*) and, at a trend level, across extended cortical areas (P^*^ < 0.05; Supplementary Table 7). No significant associations of Symbol Span score with mean MPF or CT values were found in any brain regions in the SSD group (Supplementary Table 7).

## Discussion

The current study provides novel evidence of myelin-related alterations in the cortex of patients with SSD using MPF and MPF-based NLI. Data presented here suggest decreased cortical myelin profile NLI and increased MPF with illness duration in SSD patients. NLI changes start to emerge in patients within 2.5–5.5 years since the onset of psychosis (SSD-M) near the orbitofrontal area, whereas for the longer duration patients (SSD-L), substantial NLI decreases were found in extended areas across bilateral prefrontal and anterior cingulate regions. These findings are consistent with literature suggesting progressive cortical changes in SSD patients with age and illness duration (Schnack et al. 2016; Cropley et al. 2017; Palaniyappan and Sukumar 2020). The locations of cortical changes identified here also largely coincide with results from previous histological and MRI studies of myelin abnormalities in SSD (Takahashi et al. 2011; Uranova et al. 2011; Wei et al. 2018).

A main pattern underlying observed NLI changes in the SSD-L group is increased MPF in midcortical layers, particularly in bilateral prefrontal region. Increased MPF may indicate higher myelin content in the tissue, which was a relatively surprising but robust finding in our dataset. This result may be reflective of certain complex microstructural alterations, which resonate with some existing findings in previous histological studies. In particular, Uranova et al. (2011) found substantial ultrastructural myelin and oligodendrocyte alterations in prefrontal gray matter of schizophrenia patients through postmortem tissue examination, including accumulation of unknown-origin myelin-like substance within glia, and axonal atrophy accompanied by swollen myelin sheaths and peri-axonal oligodendroglial processes. These myelin abnormalities may not necessarily lead to dramatic changes in total myelin content as measured by MRI but may result in disrupted myeloarchitectural organization and myelin profile across cortical layers. Thus, data presented here support the use of NLI as a metric sensitive to myeloarchitectural changes within the cortical ribbon and suggest that myelin abnormalities in young adult SSD patients may exist in specific cortical layers in forms other than apparent myelin loss.

Interestingly, regional variations of MPF-based NLI and its association with MPF in the HC group (Fig. 1) resonate with records from histological studies on myeloarchitectonics in the early 20th century (Vogt 1910; Braitenberg 1962; Nieuwenhuys 2013) and with recent imaging findings in healthy individuals using quantitative T_1_ mapping (Sprooten et al. 2019). We found that the prefrontal and cingulate areas showed some of the highest myelin profile nonlinearity, whereas highly myelinated regions such as those along the central sulcus and in occipital lobe had more linear profiles. This observation is particularly noteworthy because it appears to reflect the fundamental principles of cortical microstructural organization and regional function. High-order regions such as the prefrontal areas typically have a standard 6-layer cortical organization with the outer layers occupied primarily by dendritic arborization to sustain high-order functions (Sprooten et al. 2019) and therefore low on myelin content. In these regions, pyramidal cells situated in layer III and V produce the majority of myelinated fibers projecting to white matter (Nieuwenhuys 2013). These distinctively structured layers then give rise to a relative nonlinear increase of myelin content from the pial surface to white matter boundary. In contrast, unimodal cortices including the primary visual, auditory, and sensory-motor areas usually deviate from this standard organization and may have multiple layers merging together or one cell type extending to neighboring layers (Kiernan and Rajakumar 2013). The primary motor cortex, for instance, has layers II to VI consisting almost entirely of pyramidal cells (Kiernan and Rajakumar 2013). This leads to an overall higher myelin content across the cortical ribbon and a more gradual accumulation of myelinated fibers across the cortical ribbon, which results in a relatively linear myelin profile. The inherent variations in the myelin cortical profile across cortical areas observed in the current study further support the added value of NLI and highlight the significance of investigating layer-specific cortical myelin changes in clinical populations.

There have been a number of histological studies examining layer-specific myelin and oligodendrocyte-related changes in SSD, although yielding mixed results. In Brodmann area 10 located in the anterior prefrontal region, decreased density of oligodendroglia in layer V and VI has been found in chronic schizophrenia patients compared with HC (Uranova et al. 2004; Kolomeets and Uranova 2019). In contrast, other studies on glial density in layers of the prefrontal and anterior cingulate cortices noted no difference between controls and patients with schizophrenia or schizoaffective disorder (Benes et al. 1991; Hoistad et al. 2013). Recently, using T_1_w/T_2_w myelin imaging, Wei et al. (2020) reported increased myelin content in superficial and midcortical layers of inferior parietal lobe but decreased myelin in midcortical layers of insula and posterior cingulate regions in first-episode SSD. Taken together, these observations suggest that the changes in cortical myelin NLI and increased MPF found in our study may be driven by several mechanisms including abnormal myelin sheath and oligodendroglial activity (Uranova et al. 2011), increased number of neurons accompanied by reduced number of dendritic spines (Selemon et al. 2003; Wei et al. 2020), and loss or shrinkage of specific cortical layers (Wagstyl et al. 2016).

Another potential mechanism underlying increased MPF in middle layers in SSD-L is remyelination and/or other compensatory mechanisms. Remyelination could be triggered by two mechanisms in this scenario: 1) compensation for subcortical myelin deficits and network disconnection, and 2) promyelination effects of antipsychotic treatments. For chronic SSD patients, microstructural abnormalities are often observed in white matter (Parnanzone et al. 2017). These deficits degrade brain signal synchronization, which according to mechanistic models of brain development could activate compensatory mechanisms in the cortex such as increased myelination (Bartzokis 2012; Ehrlich et al. 2014). It has also been suggested that the structural changes in psychiatric disorders observed after disease onset may be considered as adaptive “cortical reorganization” instead of progressive pathology (Palaniyappan and Sukumar 2020). Therefore, increased cortical myelin may represent further neuroadaptation that occurs in response to existing deficits. As for the effect of certain medications, previous studies noted that many classes of antipsychotics and antidepressants are designed to alter neurotransmission, and therefore have neuroglial signaling effects that may influence oligodendrocyte activity and myelination (Bartzokis et al. 2007). A more recent study suggested that the promyelination effect of antipsychotics is only robust during the initial period (Tishler et al. 2018), which suggests that the interaction between pharmaceutical intervention and disease progression to be more complicated.

Notably, the finding of trend level decreased MPF in the short illness duration SSD group but increased MPF in the long-duration group is particularly intriguing. Although with cross-sectional data we are limited in our ability to infer the developmental trajectory of pathology with illness duration, this observation underlines the importance of accounting for specific disease stages in future examination of myelin changes in SSD.

The myeloarchitectural changes described by NLI also appear to relate to CT (Fig. 6) and potentially be one of the sources of CT changes in SSD, although further longitudinal studies will be needed to confirm this hypothesis. No CT changes were observed even at trend level between short-duration patients and HC, whereas for NLI and volumetric MPF, differences appeared to already emerge or be present across several brain regions. This finding might also indicate a slightly higher sensitivity of the quantitative MT-based metrics for pathological changes compared with CT in early disease stages. The biological basis of cortical thinning in SSD is still under debate. Recent studies using machine learning methods have identified anatomically distinct SSD subgroups and have suggested that the presence of cortical thinning is not universal for all schizophrenia patients but may be dependent on specific symptom combination and symptom severity (Pan et al. 2020). Previous histological studies have associated reduced CT with microstructural abnormalities including loss of dendritic spines, axonal terminals, and oligodendrocytes (Uranova et al. 2004; Garey 2010). Whereas the findings reported here cannot disentangle the differential effect of these factors, they do point to the overall direction of a more compact and homogeneous cell/fiber arrangements in the cortex of patients with SSD, an observation consistent with recent diffusion imaging reports of increased microstructural complexity in patients with chronic schizophrenia (McKenna et al. 2019).

Lastly, within the SSD group, decreased NLI appears to associate with decreased working memory capacity particularly in left caudal middle frontal area, a region consistently shown by previous functional MRI studies to activate during working memory tasks (Rottschy et al. 2012). These results bring together microstructural and behavioral aspects and show that myeloarchitectural changes are likely to impact cognitive function in SSD.

There are several limitations that need to be better addressed by future studies. A first limitation is the cross-sectional design and the relatively small sample size in each illness duration group. Despite the reasonable effect size of our results, there is still the possibility of a cohort effect where variation with illness duration may reflect differences in the sample composition rather than a change over time. Moreover, our SSD group included individuals with both schizophrenia and schizoaffective disorder, which may lead to a higher level of heterogeneity within the sample and increased sensitivity to sample-size-related problems. Therefore, longitudinal studies with larger sample sizes are essential to further delineate subtypes in patients and confirm current findings of progressive neurostructural changes. Second, partial volume averaging may have affected our results, especially in the presence of reduced cortical thickness. The consistency between cortical myelin profiles presented here and those observed in previous histological and imaging studies may indicate that our current voxel size was adequate in capturing NLI variations across regions. The finding of midcortical MPF mediating NLI changes in patients should also be robust because midcortical layers are less susceptible to partial voluming than outer layers. However, it would still be important for future studies to replicate the current findings using finer voxels, preferably under 1 mm in size. Finally, because of incomplete medication information, we were not able to adjust for the effects of antipsychotic dose and cumulative antipsychotic exposure, which have been suggested to include alterations in brain microstructure and myelination (Bartzokis et al. 2012; Tishler et al. 2018). However, it is also important to note that the apparent association between certain antipsychotic treatment and neurological changes may be biased by individual’s treatment response and therefore not necessarily reflect a causal relationship (Goff et al. 2017). Future studies in yet unmedicated first episode patients will be necessary to establish myelin status at the disease onset and discriminate medication effects on cortical myelin.

In summary, using novel quantitative MPF mapping, we have shown here that SSD are associated with altered cortical myelin profile, which may reflect, in part, increased myelin content in the midcortical layers. More importantly, these alterations appear to progress with illness duration and are related to compromised working memory capacity. Future studies are needed to confirm the current findings, preferably with a longitudinal design.

## Notes

We thank all of our participants for their help with this study and Research Match and NAMI for supporting our recruitment efforts. Part of these data was previously presented in abstract form at the Organization of Human Brain Mapping Annual Meeting (Virtual Meeting, 2020). *Conflict of Interest*: None declared.

## Funding

National Institutes of Health (R01MH108962 to M.L., R01EB027087 to A.S.).

## Supplementary Material

Revised_supplementary_tgab015
