# Supplementary material for: Quantitative Macromolecular Proton Fraction Mapping Reveals Altered Cortical Myelin Profile in Schizophrenia Spectrum Disorders
Source: Cereb Cortex Commun. 2021 Feb 24;2(2):tgab015. doi: 10.1093/texcom/tgab015 (PMC8271044; doi:10.1093/texcom/tgab015)
Supplement: Revised_supplementary_tgab015 [file revised_supplementary_tgab015.pdf]

Supplementary materials

Exclusion of enrolled participants

A total of 59 potential participants with SSD and 55 HC provided informed consent. All gave written consent to participate according to the requirements of the Institutional Review Board at New York University Grossman School of Medicine. Enrolled subjects were excluded from the study due to MRI findings indicating brain abnormalities or precluding image processing (three HC), substance abuse (five patients and three HC), history of major depression or attention deficit hyperactivity disorder (four HC), brain trauma (one HC and one patient), organic brain disorder (four patients), or not meeting SSD diagnostic criteria (three patients). MT data was not acquired in three HC and four patients due to inability to comply with the full scanning protocol. Imaging data was also not obtained for one patient who did not fit in the scanner. Four controls and eight patients were lost to follow-up. Additionally, three datasets in each group were eliminated due to presence of severe motion artifacts in MRI images, leaving data from 30 individuals in the SSD group and 34 in the HC group for analysis (Table 1).

Table S1. Medication list of patients with SSD

ID	Diagnosis	Gender	Age	Antipsychotic		Antidepressant	Anxiolytic/benzodiazepine	Mood stabilizer	Anticholinergic
				First generation	Second generation				
SSD-S1	Schizoaffective	M	29	-	Risperidone	-	-	-	-
SSD-S2	Schizoaffective	F	21	-	-	-	Clonazepam + Lorazepam		-
SSD-S3	Schizophrenia	M	22	-	Aripiprazole	-	-	-	-
SSD-S4	Schizophrenia	M	23	Haloperidol	-	-	-	-	Benztropine
SSD-S5	Schizophrenia	M	23	-	Risperidone	-	-	-	-
SSD-S6	Schizophrenia	M	23	-	-	-	-	-	-
SSD-S7	Schizophrenia	M	21	-	-	-	-	-	-
SSD-S8	Schizophrenia	M	24	-	Aripiprazole	-	-	Lithium	-
SSD-S9	Schizoaffective	M	25	-	Risperidone	Escitalopram	-	-	-
SSD-S10	Schizoaffective	F	27	-	Clozapine	Bupropion	Lorazepam	-	-
SSD-S11	Schizoaffective	F	28	-	-	Bupropion	-	Lamotrigine	-
SSD-M1	Schizoaffective	F	23	Haloperidol	-	-	-	-	-
SSD-M2	Schizoaffective	F	22	-	Aripiprazole + Quetiapine	Bupropion	Alprazolam	-	-
SSD-M3	Schizoaffective	M	24	-	Risperidone	-	-	-	-
SSD-M4	Schizoaffective	M	29	-	Aripiprazole	-	-	-	-
SSD-M5	Schizoaffective	M	22	-	Aripiprazole	-	-	-	-
SSD-M6	Schizophrenia	M	21	-	Lurasidone	-	Clonazepam	Lamotrigine	-
SSD-M7	Schizophrenia	M	25	-	Olanzapine	Escitalopram	-	-	-
SSD-M8	Schizophrenia	M	25	-	Aripiprazole	Escitalopram	-	-	-
SSD-M9	Schizophrenia	M	25	-	-	-	-	-	-
SSD-M10	Schizophrenia	M	30	-	Aripiprazole	-	-	-	-
SSD-L1	Schizoaffective	F	28	-	Clozapine	Fluoxetine	-	-	-
SSD-L2	Schizophrenia	F	24	-	Olanzapine	-	-	Gabapentin	-
SSD-L3	Schizoaffective	M	30	-	-	-	-	-	-
SSD-L4	Schizoaffective	M	25	-	Risperidone	-	-	-	-
SSD-L5	Schizophrenia	F	28	-	Olanzapine	-	-	-	-
SSD-L6	Schizoaffective	M	25	-	Risperidone	Sertraline	-	-	-
SSD-L7	Schizophrenia	F	27	-	-	-	Clonazepam	-	-
SSD-L8	Schizoaffective	F	31	-	Clozapine	-	-	Lithium	Benztropine
SSD-L9	Schizophrenia	M	29	-	Risperidone	-	-	-	-

Resolution boosted MPF

To confirm the validity of MPF-derived cortical myelin profile, we applied a self-similarity based super-resolution method on MPF maps using our high-resolution T1w MPRAGE image. With this method, we were able to introduce the anatomical details in the MPRAGE image to the MT and VFA maps and artificially increase their resolution by a factor of two. The procedure was done as follows: We first doubled the matrix size of the low-resolution images using linear interpolation. The MPRAGE image was then co-registered to the space of these interpolated images using affine transformations (FSL, FLIRT). Then, the low resolution images were re-interpolated and reconstructed by taking the weighted sum within a 7x7x7 sliding window, where the weights were calculated using the self-similarity of signal intensity in the low resolution image and MPRAGE image(1). The self-similarity between two voxels in an image was defined by a Gaussian model, with an exponent determined from the signal intensity differences, normalized by the signal mean in the brain. Finally, we imposed a constraint that the down-sampled version of reconstructed images has to be equal to the original low resolution images to ensure the data consistency (1). The self-similarity based super-resolution algorithm has been widely used in computer sciences and was validated qualitatively and quantitatively in the field of MRI (2, 3). Using the resolution-boosted images, we fitted MPF maps and calculated cortical myelin profile NLI as described in the main article. Comparison between NLI generated from the original and resolution-boosted MPF in healthy control group showed good correspondence (Figure S1).

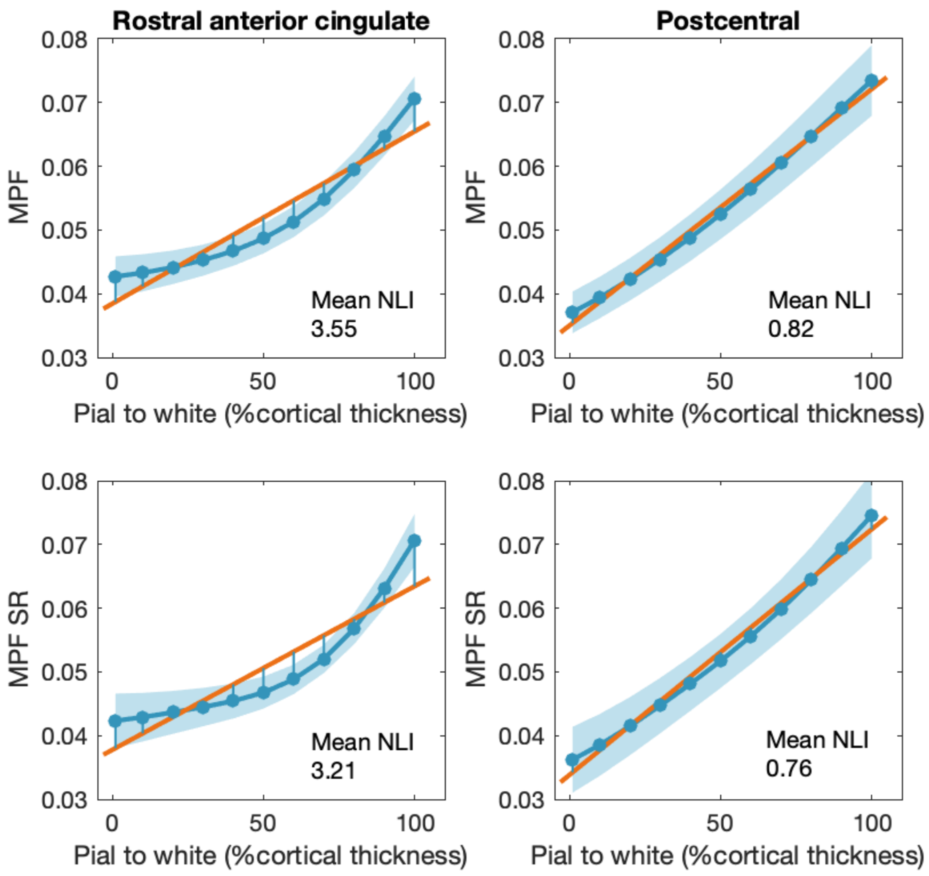

Figure S1. Within HC group, the shape of cortical myelin profile and NLI calculated from original MPF maps were comparable to those generated using super resolution method. Significant positive correlations were found between the two methods, with the Pearson's correlation coefficient for HC group of $r_{\text{original-SR}} = 0.89$ ($p<.001$). Figure shows cortical myelin profile captured by the two approaches from two representative regions, right hemisphere rostral anterior cingulate and postcentral area, with high and low NLI respectively. MPF: macromolecular proton fraction; NLI: nonlinearity index; SR: super-resolution.

Table S2. Group comparison statistics for volumetric MPF, including mean and standard deviation of MPF values for all groups, uncorrected p-value, and Cohen's d (positive values indicate patients > controls). Corresponding statistics for group comparison controlling age and gender are also included.

Region (Left/Right hemisphere)		HC		SSD-S						SSD-M						SSD-L					
		Mean	STD	Mean	STD	Group comparison		Controlling for age and gender		Mean	STD	Group comparison		Controlling for age and gender		Mean	STD	Group comparison		Controlling for age and gender	
						p-value	Cohen's d	p-value	Cohen's d			p-value	Cohen's d	p-value	Cohen's d			p-value	Cohen's d	p-value	Cohen's d
Bankssts[1]	L	.0527	.0024	.0515	.0036	.2269	-0.43	.3442	-0.33	.0513	.0036	.1704	-0.50	.2561	-0.41	.0523	.0032	.7060	-0.14	.3694	-0.34
Caudal anterior cingulate	L	.0470	.0025	.0473	.0033	.7308	0.12	.5283	0.22	.0470	.0036	.9872	0.01	.7486	0.12	.0501	.0024	.0021	1.23	.0037	1.15
Caudal middle frontal	L	.0533	.0031	.0530	.0033	.8023	-0.09	.9948	0.00	.0517	.0032	.1567	-0.52	.2250	-0.44	.0541	.0028	.5241	0.24	.7464	0.12
Cuneus	L	.0540	.0030	.0525	.0031	.1699	-0.48	.2151	-0.44	.0527	.0039	.2659	-0.41	.3432	-0.34	.0536	.0021	.7389	-0.13	.6344	-0.18
Entorhinal	L	.0517	.0047	.0506	.0055	.5248	-0.23	.4967	-0.25	.0527	.0056	.6176	0.19	.8255	0.09	.0560	.0049	.0315	0.90	.0977	0.68
Fusiform	L	.0551	.0030	.0542	.0046	.4452	-0.27	.5781	-0.2	.0541	.0028	.3890	-0.33	.4513	-0.29	.0552	.0027	.9521	0.02	.5044	-0.25
Inferior parietal	L	.0523	.0027	.0511	.0031	.2263	-0.43	.3163	-0.35	.0511	.0033	.2594	-0.41	.3539	-0.34	.0520	.0030	.7514	-0.12	.5013	-0.25
Inferior temporal	L	.0549	.0065	.0532	.0042	.4151	-0.29	.5039	-0.23	.0552	.0064	.8915	0.05	.8390	0.07	.0554	.0036	.1857	0.51	.7783	-0.11
Isthmus cingulate	L	.0506	.0027	.0496	.0033	.3039	-0.36	.4697	-0.25	.0498	.0042	.4648	-0.27	.6669	-0.16	.0518	.0022	.2279	0.46	.4135	0.31
Lateral occipital	L	.0569	.0032	.0555	.0036	.2320	-0.42	.2758	-0.38	.0560	.0036	.4701	-0.26	.4913	-0.25	.0559	.0028	.4233	-0.30	.2273	-0.46
Lateral orbitofrontal	L	.0519	.0031	.0512	.0026	.5478	-0.21	.6009	-0.18	.0507	.0036	.3039	-0.37	.3467	-0.34	.0542	.0034	.0559	0.74	.0642	0.71
Lingual	L	.0538	.0025	.0528	.0038	.3190	-0.35	.4280	-0.28	.0538	.0045	.9863	0.01	.8385	0.07	.0533	.0020	.6104	-0.19	.3652	-0.34
Medial orbitofrontal	L	.0513	.0026	.0510	.0032	.7965	-0.09	.9583	0.02	.0497	.0043	.1469	-0.53	.2154	-0.45	.0540	.0045	.0195	0.91	.0439	0.78
Middle temporal	L	.0504	.0028	.0499	.0028	.5433	-0.21	.8141	-0.08	.0500	.0038	.6718	-0.15	.8675	-0.06	.0523	.0028	.0802	0.67	.2540	0.43
Parahippocampal	L	.0505	.0038	.0492	.0029	.3192	-0.35	.4406	-0.27	.0500	.0050	.7482	-0.12	.8965	-0.05	.0521	.0042	.2792	0.41	.4350	0.30
Paracentral	L	.0581	.0034	.0573	.0043	.4951	-0.24	.6644	-0.15	.0561	.0031	.1022	-0.60	.1617	-0.51	.0581	.0043	.9975	0.00	.7349	-0.13
Pars opercularis	L	.0500	.0029	.0493	.0032	.5137	-0.23	.8034	-0.09	.0487	.0033	.2188	-0.45	.3728	-0.32	.0524	.0027	.0336	0.82	.0954	0.64
Pars orbitalis	L	.0515	.0035	.0501	.0035	.2495	-0.40	.3112	-0.36	.0498	.0037	.1748	-0.50	.2252	-0.44	.0547	.0048	.0299	0.84	.0430	0.78
Pars triangularis	L	.0508	.0027	.0495	.0034	.1856	-0.47	.2985	-0.37	.0491	.0040	.1209	-0.57	.1923	-0.48	.0547	.0035	.0010	1.33	.0034	1.16
Pericalcarine	L	.0542	.0037	.0524	.0031	.1376	-0.52	.1517	-0.51	.0537	.0029	.6907	-0.14	.7296	-0.13	.0527	.0030	.2581	-0.43	.2300	-0.46
Postcentral	L	.0546	.0031	.0528	.0032	.1046	-0.58	.1958	-0.46	.0532	.0034	.2460	-0.42	.4479	-0.28	.0543	.0034	.8142	-0.09	.4284	-0.30
Posterior cingulate	L	.0484	.0026	.0483	.0029	.9925	0.00	.8385	0.07	.0480	.0034	.7580	-0.11	.9338	-0.03	.0505	.0022	.0309	0.84	.0421	0.79
Precentral	L	.0555	.0033	.0547	.0031	.4772	-0.25	.7598	-0.11	.0545	.0029	.3808	-0.32	.6581	-0.16	.0566	.0028	.3917	0.32	.6855	0.15
Precuneus	L	.0518	.0025	.0511	.0030	.4396	-0.27	.5208	-0.22	.0504	.0033	.1541	-0.52	.2022	-0.47	.0522	.0037	.7518	0.12	.8274	0.08
Rostral anterior cingulate	L	.0476	.0022	.0465	.0036	.2333	-0.42	.2779	-0.38	.0475	.0048	.9073	-0.04	.9803	-0.01	.0504	.0034	.0040	1.14	.0062	1.08
Rostral middle frontal	L	.0510	.0024	.0500	.0037	.3223	-0.35	.4398	-0.27	.0491	.0032	.0483	-0.73	.0723	-0.66	.0527	.0023	.0589	0.73	.1144	0.60
Superior frontal	L	.0522	.0023	.0512	.0033	.2855	-0.38	.3938	-0.3	.0508	.0031	.1165	-0.58	.1666	-0.51	.0529	.0027	.4307	0.30	.6380	0.18
Superior parietal	L	.0542	.0032	.0525	.0029	.1332	-0.53	.2125	-0.44	.0527	.0036	.2101	-0.46	.3669	-0.33	.0542	.0043	.9883	-0.01	.7834	-0.10
Superior temporal	L	.0500	.0026	.0491	.0025	.3465	-0.33	.5350	-0.22	.0493	.0035	.4794	-0.26	.6324	-0.17	.0522	.0025	.0322	0.83	.0987	0.63
Supramarginal	L	.0510	.0027	.0497	.0034	.2033	-0.45	.3178	-0.35	.0501	.0031	.3492	-0.34	.5142	-0.24	.0507	.0029	.7247	-0.13	.3709	0.34
Frontal pole	L	.0503	.0045	.0500	.0067	.8969	-0.05	.9887	0.00	.0498	.0039	.7464	-0.12	.8026	-0.09	.0518	.0044	.3604	0.35	.5642	0.22
Temporal pole	L	.0501	.0054	.0512	.0057	.5521	0.21	.3683	0.32	.0475	.0061	.2260	-0.47	.3370	-0.37	.0550	.0036	.0202	0.97	.0074	1.09
Transverse temporal	L	.0523	.0030	.0528	.0030	.6157	0.18	.3472	0.33	.0514	.0039	.4393	-0.28	.6451	-0.17	.0535	.0031	.2991	0.39	.5926	0.20
Insula	L	.0488	.0030	.0483	.0027	.5924	-0.19	.8582	-0.06	.0475	.0034	.2587	-0.41	.4018	-0.30	.0498	.0022	.3482	0.36	.5963	0.20

Region (Left/Right hemisphere)		HC		SSD-S						SSD-M						SSD-L					
		Mean	STD	Mean	STD	Group comparison		Controlling for age and gender		Mean	STD	Group comparison		Controlling for age and gender		Mean	STD	Group comparison		Controlling for age and gender	
						p-value	Cohen's d	p-value	Cohen's d			p-value	Cohen's d	p-value	Cohen's d			p-value	Cohen's d	p-value	Cohen's d
Bankssts	R	.0515	.0017	.0501	.0030	.0516	-0.69	.0862	-0.61	.0507	.0028	.2649	-0.41	.3739	-0.32	.0535	.0024	.0065	1.08	.0165	0.94
Caudal anterior cingulate	R	.0475	.0023	.0469	.0039	.5718	-0.20	.6352	-0.17	.0475	.0035	.9907	0.00	.9560	0.02	.0494	.0028	.0474	0.77	.0768	0.68
Caudal middle frontal	R	.0540	.0025	.0529	.0033	.2427	-0.41	.3689	-0.32	.0521	.0035	.0626	-0.69	.1095	-0.59	.0543	.0037	.7845	0.10	.9526	-0.02
Cuneus	R	.0532	.0024	.0500	.0033	.0011	-1.21	.0016	-1.17	.0515	.0030	.0700	-0.67	.1205	-0.57	.0533	.0034	.9360	0.03	.9385	-0.03
Entorhinal	R	.0539	.0062	.0539	.0070	.9858	0.01	.9942	0.00	.0578	.0075	.1480	0.60	.2189	0.50	.0571	.0068	.2103	0.52	.3684	0.37
Fusiform	R	.0557	.0049	.0527	.0039	.0662	-0.66	.0547	-0.69	.0544	.0040	.4567	-0.28	.3869	-0.33	.0542	.0030	.3710	-0.34	.4097	-0.31
Inferior parietal	R	.0511	.0024	.0494	.0026	.0536	-0.69	.0759	-0.63	.0498	.0026	.1256	-0.56	.1847	-0.49	.0518	.0026	.4780	0.27	.5383	0.23
Inferior temporal	R	.0535	.0029	.0521	.0039	.1970	-0.46	.2316	-0.42	.0530	.0036	.6296	-0.18	.6001	-0.20	.0557	.0038	.0708	0.70	.1497	0.55
Isthmus cingulate	R	.0506	.0025	.0484	.0036	.0264	-0.80	.0439	-0.72	.0502	.0032	.7085	-0.14	.8886	-0.05	.0525	.0027	.0505	0.76	.0943	0.64
Lateral occipital	R	.0554	.0030	.0536	.0024	.0779	-0.63	.0806	-0.62	.0542	.0026	.2619	-0.41	.2575	-0.41	.0553	.0029	.9467	-0.03	.8942	-0.05
Lateral orbitofrontal	R	.0526	.0031	.0525	.0045	.9489	-0.02	.9810	0.01	.0524	.0044	.8448	-0.07	.8736	-0.06	.0546	.0039	.1154	0.60	.1703	0.52
Lingual	R	.0529	.0028	.0509	.0037	.0649	-0.66	.0783	-0.63	.0527	.0046	.8756	-0.06	.9390	-0.03	.0533	.0025	.7110	0.14	.8119	0.09
Medial orbitofrontal	R	.0509	.0023	.0521	.0045	.2632	0.39	.2576	0.40	.0509	.0049	.9874	-0.01	.9967	0.00	.0556	.0045	.0001*	1.63	.0001*	1.63
Middle temporal	R	.0504	.0022	.0493	.0028	.1744	-0.48	.2645	-0.39	.0501	.0029	.7168	-0.13	.8632	-0.06	.0529	.0015	.0029	1.19	.0118	0.99
Parahippocampal	R	.0522	.0058	.0482	.0030	.0351	-0.75	.0272	-0.79	.0507	.0045	.4723	-0.27	.3291	-0.37	.0510	.0035	.5497	-0.23	.4278	-0.30
Paracentral	R	.0580	.0033	.0561	.0039	.1045	-0.58	.1948	-0.46	.0557	.0035	.0539	-0.71	.1077	-0.59	.0590	.0056	.5068	0.25	.7404	0.13
Pars opercularis	R	.0502	.0024	.0487	.0035	.0929	-0.60	.1479	-0.51	.0501	.0028	.8674	-0.06	.9168	0.04	.0516	.0028	.1503	0.55	.2676	0.42
Pars orbitalis	R	.0516	.0046	.0504	.0046	.4548	-0.26	.5144	-0.23	.0501	.0039	.3805	-0.32	.4235	-0.29	.0521	.0043	.7555	0.12	.8858	0.05
Pars triangularis	R	.0514	.0027	.0504	.0032	.3402	-0.33	.4340	-0.27	.0511	.0039	.7693	-0.11	.8772	-0.06	.0527	.0032	.1943	0.49	.2985	0.39
Pericalcarine	R	.0529	.0035	.0500	.0027	.0152	-0.88	.0197	-0.84	.0511	.0042	.1746	-0.50	.2042	-0.46	.0525	.0028	.7249	-0.13	.6455	-0.17
Postcentral	R	.0547	.0023	.0530	.0032	.0552	-0.68	.0847	-0.61	.0536	.0028	.1946	-0.47	.3356	-0.35	.0553	.0032	.5528	0.22	.5959	0.20
Posterior cingulate	R	.0488	.0024	.0479	.0035	.3441	-0.33	.4655	-0.26	.0485	.0032	.7626	-0.11	.9404	-0.03	.0510	.0029	.0255	0.87	.0480	0.76
Precentral	R	.0561	.0028	.0547	.0040	.2040	-0.45	.3244	-0.35	.0552	.0026	.3677	-0.33	.5930	-0.19	.0570	.0032	.3698	0.34	.5740	0.21
Precuneus	R	.0518	.0025	.0495	.0034	.0215	-0.83	.0342	-0.76	.0503	.0028	.1152	-0.58	.1791	-0.49	.0525	.0034	.4589	0.28	.5855	0.21
Rostral anterior cingulate	R	.0480	.0024	.0474	.0036	.5244	-0.22	.5820	-0.19	.0477	.0043	.7987	-0.09	.8873	-0.05	.0493	.0033	.2051	0.48	.2202	0.47
Rostral middle frontal	R	.0519	.0026	.0512	.0037	.5138	-0.23	.6419	-0.16	.0508	.0046	.3296	-0.35	.3985	-0.31	.0536	.0033	.1062	0.62	.2047	0.48
Superior frontal	R	.0527	.0021	.0520	.0035	.4205	-0.28	.4900	-0.24	.0512	.0033	.0887	-0.63	.1060	-0.59	.0537	.0026	.2442	0.44	.3574	0.35
Superior parietal	R	.0535	.0029	.0513	.0036	.0479	-0.71	.0745	-0.63	.0519	.0028	.1361	-0.55	.2257	-0.44	.0546	.0036	.3346	0.37	.3867	0.33
Superior temporal	R	.0505	.0023	.0487	.0031	.0427	-0.72	.0728	-0.64	.0500	.0033	.6267	-0.18	.7790	-0.10	.0525	.0022	.0258	0.87	.0736	0.69
Supramarginal	R	.0510	.0022	.0495	.0034	.0891	-0.60	.1286	-0.54	.0499	.0027	.2140	-0.45	.3283	-0.36	.0519	.0023	.2769	0.41	.3306	0.37
Frontal pole	R	.0510	.0039	.0490	.0054	.1856	-0.47	.2305	-0.42	.0506	.0047	.7901	-0.10	.8793	-0.05	.0530	.0040	.1894	0.50	.2825	0.41
Temporal pole	R	.0494	.0044	.0523	.0069	.1295	0.57	.1416	0.55	.0507	.0042	.4738	0.28	.5301	0.24	.0534	.0060	.0454	0.84	.0467	0.84
Transverse temporal	R	.0530	.0028	.0517	.0032	.2287	-0.42	.3886	-0.30	.0521	.0047	.4515	-0.27	.6215	-0.18	.0545	.0034	.1751	0.52	.4280	0.30
Insula	R	.0495	.0025	.0478	.0038	.0996	-0.58	.1644	-0.49	.0481	.0034	.1571	-0.52	.2652	-0.41	.0501	.0037	.5648	0.22	.9307	0.03

[1]Banks of the superior temporal sulcus

*Significant after Bonferroni correction

Table S3. Group comparison statistics for CT, including mean and standard deviation of CT values for all groups, uncorrected p-value, and Cohen's d (positive values indicate patients > controls). Corresponding statistics for group comparison controlling age and gender are also included.

Region (Left/Right hemisphere)		HC		SSD-S						SSD-M						SSD-L					
		Mean	STD	Mean	STD	Group comparison		Controlling for age and gender		Mean	STD	Group comparison		Controlling for age and gender		Mean	STD	Group comparison		Controlling for age and gender	
						p-value	Cohen's d	p-value	Cohen's d			p-value	Cohen's d	p-value	Cohen's d			p-value	Cohen's d	p-value	Cohen's d
Bankssts[1]	L	2.74	0.14	2.68	0.12	.1908	-0.46	.1549	-0.50	2.77	0.10	.5416	0.22	.5818	0.20	2.64	0.13	.0614	-0.72	.1011	-0.63
Caudal anterior cingulate	L	2.89	0.23	2.78	0.17	.1565	-0.50	.1481	-0.51	2.88	0.28	.9281	-0.03	.8638	-0.06	2.67	0.22	.0171	-0.93	.0070	-1.06
Caudal middle frontal	L	2.92	0.14	2.87	0.14	.2871	-0.37	.1577	-0.50	2.86	0.08	.1835	-0.49	.1253	-0.56	2.67	0.18	<.0001*	-1.76	.0001*	-1.57
Cuneus	L	2.04	0.12	2.05	0.10	.7558	0.11	.6407	0.16	1.98	0.14	.2129	-0.45	.3157	-0.37	1.91	0.14	.0076	-1.05	.0060	-1.09
Entorhinal	L	3.54	0.24	3.53	0.25	.8896	-0.05	.6730	-0.15	3.59	0.34	.6429	0.17	.7507	0.12	3.47	0.30	.4289	-0.30	.7738	-0.11
Fusiform	L	3.02	0.09	2.99	0.09	.2484	-0.41	.2048	-0.45	3.00	0.11	.5047	-0.24	.4523	-0.27	2.89	0.18	.0029	-1.19	.0049	-1.11
Inferior parietal	L	2.71	0.11	2.70	0.12	.7690	-0.10	.6609	-0.15	2.71	0.07	.9601	-0.02	.8716	-0.06	2.57	0.10	.0015	-1.28	.0039	-1.15
Inferior temporal	L	3.09	0.14	3.06	0.12	.5223	-0.22	.2881	-0.37	3.13	0.14	.4051	0.30	.6682	0.16	2.95	0.23	.0350	-0.82	.0935	-0.64
Isthmus cingulate	L	2.56	0.18	2.56	0.15	.9030	0.04	.9460	0.02	2.58	0.22	.7557	0.11	.8473	0.07	2.53	0.15	.6622	-0.16	.6159	-0.19
Lateral occipital	L	2.31	0.13	2.31	0.13	.9695	0.01	.9373	0.03	2.29	0.13	.7685	-0.11	.8119	-0.09	2.22	0.11	.0636	-0.71	.0593	-0.73
Lateral orbitofrontal	L	3.07	0.15	3.02	0.11	.3585	-0.32	.1405	-0.52	3.00	0.15	.1980	-0.47	.0511	-0.72	2.90	0.19	.0078	-1.05	.0153	-0.95
Lingual	L	2.18	0.12	2.18	0.11	.9757	-0.01	.8391	-0.07	2.18	0.10	.8995	-0.05	.8338	-0.08	2.07	0.21	.0461	-0.77	.0858	-0.66
Medial orbitofrontal	L	2.89	0.21	2.81	0.27	.3591	-0.32	.2240	-0.43	2.69	0.25	.0163	-0.90	.0066	-1.03	2.48	0.20	<.0001*	-1.96	<.0001*	-1.87
Middle temporal	L	3.15	0.13	3.13	0.12	.6370	-0.16	.3174	-0.35	3.14	0.14	.9005	-0.05	.5614	-0.21	3.05	0.14	.0528	-0.75	.1906	-0.50
Parahippocampal	L	2.93	0.37	2.77	0.26	.1957	-0.46	.2865	-0.37	2.95	0.17	.8600	0.06	.6481	0.17	2.80	0.34	.3404	-0.36	.1529	-0.55
Paracentral	L	2.60	0.17	2.54	0.14	.3102	-0.36	.2661	-0.39	2.63	0.18	.6371	0.17	.6153	0.18	2.51	0.11	.1180	-0.60	.2097	-0.48
Pars opercularis	L	2.93	0.11	2.90	0.13	.4307	-0.28	.1587	-0.50	2.89	0.07	.2440	-0.43	.0998	-0.61	2.74	0.19	.0004*	-1.46	.0018	-1.25
Pars orbitalis	L	3.08	0.17	3.11	0.23	.6339	0.17	.9320	0.03	3.01	0.12	.2395	-0.43	.0953	-0.61	2.84	0.18	.0005*	-1.41	.0028	-1.19
Pars triangularis	L	2.76	0.13	2.76	0.19	.9138	0.04	.6272	-0.17	2.67	0.15	.0776	-0.65	.0153	-0.91	2.54	0.11	<.0001*	-1.72	.0002	-1.54
Pericalcarine	L	1.86	0.16	1.78	0.13	.1334	-0.53	.1213	-0.55	1.84	0.13	.7655	-0.11	.7676	-0.11	1.74	0.12	.0418	-0.79	.0590	-0.73
Postcentral	L	2.28	0.11	2.24	0.13	.3179	-0.35	.2494	-0.40	2.27	0.10	.7601	-0.11	.6748	-0.15	2.19	0.12	.0363	-0.81	.0646	-0.71
Posterior cingulate	L	2.69	0.12	2.67	0.08	.5457	-0.21	.3852	-0.30	2.66	0.18	.4877	-0.25	.3509	-0.34	2.61	0.17	.1023	-0.63	.1578	-0.54
Precentral	L	2.86	0.13	2.79	0.11	.0930	-0.60	.0609	-0.67	2.83	0.10	.4270	-0.29	.3798	-0.32	2.76	0.11	.0478	-0.76	.1015	-0.63
Precuneus	L	2.58	0.09	2.59	0.09	.8728	0.06	.9351	0.03	2.57	0.10	.6425	-0.17	.6388	-0.17	2.46	0.10	.0011	-1.31	.0023	-1.22
Rostral anterior cingulate	L	3.26	0.17	3.16	0.20	.1068	-0.57	.0402	-0.73	3.28	0.35	.8199	0.08	.9374	-0.03	2.90	0.20	<.0001*	-2.09	<.0001*	-1.94
Rostral middle frontal	L	2.75	0.12	2.71	0.21	.4544	-0.26	.1685	-0.49	2.66	0.09	.0369	-0.78	.0037	-1.11	2.50	0.15	<.0001*	-1.97	<.0001*	-1.78
Superior frontal	L	3.08	0.13	3.05	0.16	.5252	-0.22	.2935	-0.37	3.02	0.14	.2198	-0.45	.1239	-0.56	2.86	0.11	.0001	-1.65	.0005	-1.41
Superior parietal	L	2.36	0.11	2.36	0.11	.9869	0.01	.9838	-0.01	2.33	0.07	.5440	-0.22	.5652	-0.21	2.27	0.11	.0499	-0.76	.0711	-0.69
Superior temporal	L	3.02	0.14	3.03	0.19	.8437	0.07	.8213	-0.08	3.03	0.13	.9425	0.03	.6010	-0.19	3.01	0.16	.8077	-0.09	.8935	-0.05
Supramarginal	L	2.78	0.11	2.79	0.11	.8414	0.07	.7628	-0.11	2.80	0.12	.5692	0.21	.8692	0.06	2.70	0.06	.0495	-0.76	.1982	-0.49
Frontal pole	L	3.06	0.24	2.94	0.34	.1969	-0.45	.0863	-0.61	2.95	0.30	.2391	-0.43	.1183	-0.57	2.94	0.41	.2552	-0.43	.5332	-0.24
Temporal pole	L	3.84	0.23	3.99	0.17	.0577	0.68	.1020	0.58	4.00	0.23	.0704	0.67	.1297	0.56	3.88	0.20	.6519	0.17	.4771	0.27
Transverse temporal	L	2.76	0.20	2.74	0.21	.7410	-0.12	.5179	-0.23	2.68	0.17	.2225	-0.45	.1241	-0.56	2.56	0.16	.0063	-1.08	.0157	-0.95
Insula	L	3.40	0.13	3.35	0.19	.3245	-0.35	.1875	-0.46	3.30	0.09	.0286	-0.82	.0082	-1.00	3.26	0.11	.0033	-1.17	.0057	-1.09

Region (Left/Right hemisphere)		HC		SSD-S						SSD-M						SSD-L					
		Mean	STD	Mean	STD	Group comparison		Controlling for age and gender		Mean	STD	Group comparison		Controlling for age and gender		Mean	STD	Group comparison		Controlling for age and gender	
						p-value	Cohen's d	p-value	Cohen's d			p-value	Cohen's d	p-value	Cohen's d			p-value	Cohen's d	p-value	Cohen's d
Bankssts	R	2.83	0.16	2.78	0.13	.3269	-0.34	.2203	-0.43	2.82	0.25	.9425	-0.03	.7599	-0.11	2.71	0.15	.0540	-0.74	.0816	-0.67
Caudal anterior cingulate	R	2.69	0.20	2.69	0.13	.9716	0.01	.8603	-0.06	2.65	0.34	.7163	-0.13	.5504	-0.22	2.44	0.21	.0022	-1.22	.0024	-1.21
Caudal middle frontal	R	2.93	0.14	2.83	0.18	.0621	-0.66	.0226	-0.82	2.84	0.17	.0877	-0.63	.0395	-0.76	2.66	0.17	<.0001*	-1.82	.0001	-1.64
Cuneus	R	2.05	0.13	2.05	0.13	.9952	0.00	.9907	0.00	1.98	0.08	.1438	-0.54	.1728	-0.50	1.97	0.17	.1137	-0.61	.1626	-0.53
Entorhinal	R	3.68	0.24	3.75	0.33	.4629	0.26	.7081	0.13	3.59	0.27	.3172	-0.36	.1224	-0.57	3.70	0.39	.8236	0.08	.5006	0.25
Fusiform	R	3.00	0.12	2.99	0.09	.7896	-0.09	.6461	-0.16	2.97	0.17	.5088	-0.24	.3829	-0.32	2.88	0.16	.0170	-0.93	.0254	-0.87
Inferior parietal	R	2.75	0.10	2.72	0.12	.3944	-0.30	.3984	-0.30	2.74	0.18	.7787	-0.10	.8276	-0.08	2.68	0.14	.0669	-0.71	.0828	-0.67
Inferior temporal	R	3.10	0.14	3.09	0.11	.8479	-0.07	.5807	-0.19	3.14	0.14	.3971	0.31	.5804	0.20	2.97	0.19	.0255	-0.87	.0709	-0.70
Isthmus cingulate	R	2.51	0.21	2.60	0.13	.1974	0.45	.2689	0.39	2.48	0.16	.6941	-0.14	.4710	-0.26	2.44	0.16	.3368	-0.36	.3875	-0.33
Lateral occipital	R	2.37	0.13	2.34	0.15	.5514	-0.21	.6094	-0.18	2.30	0.12	.1290	-0.56	.1569	-0.52	2.30	0.11	.1190	-0.60	.0960	-0.64
Lateral orbitofrontal	R	3.16	0.14	3.08	0.22	.1654	-0.49	.1029	-0.58	2.97	0.20	.0017	-1.21	.0006	-1.35	2.82	0.20	<.0001*	-2.23	<.0001*	-2.18
Lingual	R	2.19	0.12	2.21	0.14	.5614	0.20	.6301	0.17	2.20	0.14	.8217	0.08	.7953	0.09	2.07	0.18	.0274	-0.86	.0584	-0.73
Medial orbitofrontal	R	3.06	0.19	2.93	0.30	.1138	-0.56	.0765	-0.63	2.75	0.27	.0002	-1.48	.0001	-1.57	2.42	0.23	<.0001*	-3.21	<.0001*	-3.06
Middle temporal	R	3.19	0.15	3.10	0.14	.0893	-0.60	.0340	-0.76	3.15	0.17	.5045	-0.24	.2727	-0.40	3.05	0.12	.0094	-1.02	.0257	-0.87
Parahippocampal	R	2.87	0.25	2.79	0.29	.3631	-0.32	.4121	-0.29	2.91	0.22	.6811	0.15	.5768	0.20	2.80	0.26	.4642	-0.28	.4147	-0.31
Paracentral	R	2.63	0.10	2.58	0.17	.2339	-0.42	.1757	-0.48	2.62	0.23	.8261	-0.08	.8106	-0.09	2.55	0.13	.0522	-0.75	.1081	-0.62
Pars opercularis	R	2.93	0.15	2.94	0.18	.9436	0.02	.6985	-0.14	2.85	0.11	.1144	-0.58	.0390	-0.77	2.73	0.21	.0025	-1.21	.0095	-1.02
Pars orbitalis	R	3.06	0.18	3.09	0.21	.7049	0.13	.8747	0.06	3.01	0.20	.4435	-0.28	.3870	-0.31	2.81	0.25	.0014	-1.28	.0053	-1.11
Pars triangularis	R	2.83	0.15	2.75	0.16	.1328	-0.53	.0218	-0.83	2.73	0.23	.1005	-0.60	.0207	-0.86	2.59	0.15	<.0001*	-1.62	.0002	-1.52
Pericalcarine	R	1.80	0.13	1.74	0.17	.2353	-0.42	.2963	-0.37	1.77	0.17	.5185	-0.23	.6683	-0.16	1.73	0.15	.1749	-0.52	.1598	-0.54
Postcentral	R	2.27	0.10	2.25	0.15	.6280	-0.17	.5173	-0.23	2.25	0.17	.5846	-0.20	.4888	-0.25	2.16	0.13	.0084	-1.04	.0159	-0.94
Posterior cingulate	R	2.69	0.12	2.66	0.15	.5904	-0.19	.4223	-0.28	2.65	0.16	.3577	-0.33	.1955	-0.47	2.55	0.11	.0032	-1.17	.0033	-1.17
Precentral	R	2.81	0.15	2.77	0.14	.3770	-0.31	.3145	-0.35	2.82	0.19	.9658	0.02	.9557	-0.02	2.74	0.10	.1896	-0.50	.2637	-0.42
Precuneus	R	2.58	0.08	2.52	0.11	.0558	-0.68	.0416	-0.73	2.58	0.14	.9697	-0.01	.9346	-0.03	2.48	0.10	.0037	-1.15	.0085	-1.04
Rostral anterior cingulate	R	3.22	0.23	3.14	0.36	.4098	-0.29	.3578	-0.32	3.16	0.30	.4911	-0.25	.4450	-0.28	2.93	0.21	.0013	-1.29	.0028	-1.19
Rostral middle frontal	R	2.76	0.13	2.69	0.27	.2604	-0.40	.1319	-0.53	2.62	0.21	.0110	-0.96	.0040	-1.10	2.44	0.18	<.0001*	-2.24	<.0001*	-2.00
Superior frontal	R	3.10	0.12	3.04	0.22	.2685	-0.39	.1304	-0.53	3.00	0.18	.0435	-0.75	.0227	-0.85	2.85	0.19	<.0001*	-1.81	.0002	-1.54
Superior parietal	R	2.34	0.11	2.33	0.14	.9264	-0.03	.9512	0.02	2.34	0.16	.9197	0.04	.8155	0.08	2.30	0.09	.3197	-0.38	.1987	-0.49
Superior temporal	R	3.05	0.12	3.01	0.19	.4257	-0.28	.1422	-0.52	3.05	0.22	.9874	-0.01	.4614	-0.27	3.04	0.15	.9589	-0.02	.5688	0.22
Supramarginal	R	2.80	0.12	2.75	0.14	.1810	-0.47	.1129	-0.56	2.80	0.22	.9360	-0.03	.7947	-0.09	2.68	0.09	.0039	-1.15	.0112	-1.00
Frontal pole	R	3.08	0.23	3.00	0.37	.3914	-0.30	.2558	-0.40	3.03	0.40	.5716	-0.21	.4588	-0.27	2.89	0.17	.0223	-0.89	.0876	-0.66
Temporal pole	R	4.09	0.21	4.10	0.24	.8844	0.05	.9591	-0.02	3.94	0.27	.0768	-0.65	.0545	-0.71	4.16	0.20	.3987	0.32	.2280	0.46
Transverse temporal	R	2.80	0.19	2.76	0.37	.6742	-0.15	.4725	-0.25	2.72	0.17	.2324	-0.44	.1013	-0.60	2.61	0.18	.0127	-0.98	.0313	-0.84
Insula	R	3.35	0.14	3.36	0.16	.7834	0.10	.8991	-0.04	3.23	0.18	.0327	-0.79	.0082	-1.00	3.22	0.20	.0265	-0.86	.0464	-0.77

[1]Banks of the superior temporal sulcus

*Significant after Bonferroni correction

Table S4. Group comparison statistics for cortical myelin NLI, including mean and standard deviation of NLI values for all groups, uncorrected p-value, and Cohen's d (positive values indicate patients > controls). Corresponding statistics for group comparison controlling age and gender are also included.

Region (Left/Right hemisphere)		HC		SSD-S						SSD-M						SSD-L					
		Mean	STD	Mean	STD	Group comparison		Controlling for age and gender		Mean	STD	Group comparison		Controlling for age and gender		Mean	STD	Group comparison		Controlling for age and gender	
						p-value	Cohen's d	p-value	Cohen's d			p-value	Cohen's d	p-value	Cohen's d			p-value	Cohen's d	p-value	Cohen's d
Bankssts[1]	L	2.98	0.56	2.84	0.41	.4326	-0.27	.3621	-0.32	2.84	0.51	.4775	-0.26	.4541	-0.27	2.38	0.40	.0044	-1.13	.0097	-1.02
Caudal anterior cingulate	L	3.72	0.60	3.37	0.54	.0972	-0.59	.0708	-0.64	3.55	0.92	.5047	-0.24	.4437	-0.28	2.89	0.77	.0013	-1.29	.0037	-1.15
Caudal middle frontal	L	2.05	0.59	2.03	0.52	.9030	-0.04	.5903	-0.19	1.81	0.40	.2355	-0.43	.1217	-0.57	1.39	0.50	.0037	-1.15	.0144	-0.96
Cuneus	L	1.10	0.37	1.16	0.30	.6545	0.16	.7389	0.12	0.88	0.49	.1365	-0.55	.1289	-0.56	0.74	0.37	.0131	-0.97	.0252	-0.87
Entorhinal	L	2.72	1.73	2.87	1.15	.7933	0.09	.7318	0.12	1.93	1.08	.2042	-0.49	.2772	-0.42	2.32	0.77	.5341	-0.25	.5939	-0.21
Fusiform	L	2.94	0.44	2.83	0.42	.4630	-0.26	.3941	-0.30	2.64	0.89	.1552	-0.53	.1157	-0.58	2.45	0.55	.0083	-1.05	.0092	-1.03
Inferior parietal	L	2.00	0.54	2.08	0.65	.6650	0.15	.8285	0.08	1.82	0.45	.3609	-0.33	.2664	-0.41	1.37	0.23	.0015	-1.28	.0042	-1.14
Inferior temporal	L	3.02	0.73	2.87	0.50	.5199	-0.23	.4255	-0.28	2.87	0.41	.5183	-0.23	.3864	-0.31	2.54	0.79	.0918	-0.65	.0932	-0.64
Isthmus cingulate	L	2.49	0.40	2.43	0.28	.6756	-0.15	.5678	-0.20	2.31	0.50	.2387	-0.43	.1982	-0.47	2.27	0.51	.1707	-0.52	.2534	-0.43
Lateral occipital	L	1.70	0.58	1.77	0.56	.7169	0.13	.8702	0.06	1.56	0.49	.5004	-0.24	.4046	-0.30	1.36	0.33	.1068	-0.62	.1967	-0.49
Lateral orbitofrontal	L	3.10	0.56	2.77	0.58	.0913	-0.60	.0378	-0.74	2.67	0.62	.0400	-0.76	.0119	-0.95	2.07	0.63	<.0001*	-1.80	.0001*	-1.65
Lingual	L	1.60	0.43	1.60	0.42	.9933	0.00	.8333	-0.07	1.31	0.29	.0516	-0.72	.0334	-0.79	1.24	0.43	.0329	-0.83	.0638	-0.71
Medial orbitofrontal	L	2.65	0.52	2.23	0.66	.0321	-0.77	.0264	-0.80	2.15	0.90	.0275	-0.82	.0232	-0.85	1.31	0.85	<.0001*	-2.25	<.0001*	-2.09
Middle temporal	L	2.45	0.47	2.50	0.34	.7421	0.11	.9393	0.03	2.32	0.45	.4491	-0.27	.3475	-0.34	2.14	0.67	.1180	-0.60	.2153	-0.47
Parahippocampal	L	2.57	0.97	2.60	0.71	.9143	0.04	.6949	-0.14	2.67	0.71	.7440	0.12	.5436	0.22	2.08	0.77	.1692	-0.52	.0766	-0.68
Paracentral	L	1.13	0.59	1.09	0.50	.8409	-0.07	.7180	-0.13	0.85	0.85	.2438	-0.43	.2250	-0.44	0.86	0.38	.2044	-0.48	.3665	-0.34
Pars opercularis	L	2.15	0.48	1.93	0.42	.1772	-0.48	.0798	-0.62	1.84	0.24	.0560	-0.71	.0248	-0.84	1.46	0.60	.0007*	-1.38	.0018	-1.25
Pars orbitalis	L	2.24	0.76	2.20	1.01	.8914	-0.05	.6148	-0.18	1.70	0.42	.0392	-0.77	.0066	-1.03	0.89	0.58	<.0001*	-1.85	<.0001*	-1.79
Pars triangularis	L	2.18	0.52	1.82	0.60	.0663	-0.65	.0142	-0.89	1.87	0.55	.1167	-0.58	.0375	-0.77	1.19	0.70	<.0001*	-1.75	.0001*	-1.59
Pericalcarine	L	0.72	0.35	0.71	0.33	.9670	-0.01	.8780	-0.05	0.62	0.53	.4984	-0.25	.4306	-0.29	0.54	0.25	.1729	-0.52	.2123	-0.47
Postcentral	L	0.78	0.43	0.79	0.39	.9531	0.02	.7388	-0.12	0.70	0.46	.5843	-0.20	.3837	-0.32	0.47	0.26	.0408	-0.79	.1195	-0.60
Posterior cingulate	L	2.83	0.43	2.72	0.32	.4472	-0.27	.4099	-0.29	2.54	0.50	.0864	-0.63	.0779	-0.65	2.46	0.42	.0259	-0.87	.0312	-0.84
Precentral	L	1.11	0.56	0.91	0.51	.3025	-0.36	.1812	-0.47	0.78	0.38	.0907	-0.62	.0618	-0.69	0.68	0.42	.0359	-0.81	.0890	-0.65
Precuneus	L	1.94	0.43	1.95	0.40	.9434	0.02	.9317	-0.03	1.74	0.49	.2004	-0.47	.1761	-0.49	1.42	0.34	.0016	-1.27	.0040	-1.14
Rostral anterior cingulate	L	3.71	0.65	3.30	0.42	.0607	-0.67	.0430	-0.72	3.38	0.89	.2045	-0.46	.1774	-0.49	2.71	0.54	.0001*	-1.59	.0004*	-1.45
Rostral middle frontal	L	2.22	0.49	1.96	0.67	.1732	-0.48	.0809	-0.62	1.90	0.38	.0669	-0.68	.0272	-0.82	1.25	0.72	<.0001*	-1.77	.0001*	-1.59
Superior frontal	L	2.37	0.56	2.21	0.51	.4023	-0.29	.2255	-0.43	2.04	0.51	.1051	-0.60	.0555	-0.71	1.64	0.56	.0013	-1.29	.0050	-1.11
Superior parietal	L	1.12	0.48	1.38	0.39	.1133	0.56	.1906	0.46	1.08	0.50	.7971	-0.09	.6526	-0.16	0.66	0.43	.0117	-0.99	.0371	-0.81
Superior temporal	L	2.10	0.40	1.89	0.43	.1414	-0.52	.0964	-0.59	1.97	0.28	.3648	-0.33	.2661	-0.41	1.86	0.55	.1498	-0.55	.2197	-0.47
Supramarginal	L	1.95	0.47	2.09	0.38	.3676	0.32	.6095	0.18	1.92	0.52	.8822	-0.05	.6324	-0.17	1.60	0.43	.0499	-0.76	.1336	-0.57
Frontal pole	L	1.57	0.83	1.57	0.95	.9906	0.00	.7238	-0.12	1.28	0.71	.3255	-0.36	.1642	-0.51	1.02	1.39	.1389	-0.57	.2596	-0.43
Temporal pole	L	2.09	1.11	2.08	1.41	.9658	-0.02	.8764	0.06	2.46	0.91	.3700	0.34	.2815	0.42	3.02	2.16	.0904	0.67	.1351	0.59
Transverse temporal	L	1.43	0.65	0.94	0.48	.0274	-0.79	.0112	-0.92	1.11	0.60	.1760	-0.50	.1203	-0.57	1.11	0.53	.1831	-0.51	.3458	-0.36
Insula	L	1.89	0.42	1.67	0.48	.1600	-0.50	.1152	-0.56	1.63	0.56	.1314	-0.55	.1059	-0.59	1.33	0.42	.0011	-1.32	.0033	-1.17

Region (Left/Right hemisphere)		HC		SSD-S						SSD-M						SSD-L					
		Mean	STD	Mean	STD	Group comparison		Controlling for age and gender		Mean	STD	Group comparison		Controlling for age and gender		Mean	STD	Group comparison		Controlling for age and gender	
						p-value	Cohen's d	p-value	Cohen's d			p-value	Cohen's d	p-value	Cohen's d			p-value	Cohen's d	p-value	Cohen's d
Bankssts	R	2.80	0.56	2.89	0.62	.6374	0.16	.8411	0.07	2.59	0.68	.3337	-0.35	.2183	-0.45	2.41	0.55	.0682	-0.70	.1307	-0.58
Caudal anterior cingulate	R	3.35	0.62	3.41	0.68	.7675	0.10	.8447	0.07	3.03	0.76	.1934	-0.48	.1867	-0.48	2.45	0.74	.0006*	-1.39	.0015	-1.27
Caudal middle frontal	R	2.09	0.67	1.96	0.41	.5573	-0.21	.2464	-0.41	1.74	0.62	.1563	-0.52	.0560	-0.71	1.39	0.65	.0076	-1.05	.0293	-0.85
Cuneus	R	1.10	0.41	0.96	0.41	.3240	-0.35	.2956	-0.37	1.01	0.40	.5294	-0.23	.5522	-0.22	0.65	0.43	.0056	-1.10	.0089	-1.03
Entorhinal	R	2.87	1.67	3.28	1.31	.4638	0.26	.7022	0.14	3.12	2.18	.7197	0.14	.8669	-0.07	3.43	2.44	.4575	0.31	.4917	0.28
Fusiform	R	2.76	0.66	2.86	0.46	.6735	0.15	.6646	0.15	2.72	0.58	.8492	-0.07	.9340	-0.03	2.43	0.77	.2015	-0.49	.2439	-0.45
Inferior parietal	R	1.99	0.44	2.01	0.57	.8767	0.05	.9165	-0.04	1.88	0.52	.5097	-0.24	.3817	-0.32	1.52	0.25	.0045	-1.13	.0155	-0.95
Inferior temporal	R	2.88	0.43	2.79	0.55	.6012	-0.18	.4988	-0.24	2.73	0.87	.4890	-0.26	.3860	-0.33	2.53	0.70	.0742	-0.69	.1000	-0.63
Isthmus cingulate	R	2.35	0.39	2.33	0.33	.9051	-0.04	.7235	-0.12	2.13	0.54	.1601	-0.51	.0874	-0.63	2.15	0.36	.1823	-0.51	.2257	-0.46
Lateral occipital	R	1.76	0.48	1.78	0.57	.9210	0.03	.9198	-0.04	1.43	0.41	.0561	-0.71	.0369	-0.78	1.32	0.39	.0148	-0.95	.0318	-0.83
Lateral orbitofrontal	R	3.35	0.53	2.88	0.77	.0282	-0.79	.0235	-0.81	2.74	1.02	.0143	-0.92	.0120	-0.95	2.19	0.81	<.0001*	-1.95	<.0001*	-1.78
Lingual	R	1.56	0.36	1.48	0.29	.5070	-0.23	.3788	-0.31	1.13	0.59	.0076	-1.01	.0052	-1.06	1.11	0.31	.0017	-1.26	.0037	-1.16
Medial orbitofrontal	R	3.35	0.52	2.76	1.00	.0148	-0.88	.0139	-0.89	2.50	0.74	.0002*	-1.47	.0002*	-1.48	1.52	0.96	<.0001*	-2.90	<.0001*	-2.73
Middle temporal	R	2.40	0.65	2.41	0.53	.9430	0.02	.8934	-0.05	2.18	0.58	.3593	-0.33	.2746	-0.40	2.07	0.74	.2003	-0.49	.3135	-0.38
Parahippocampal	R	2.69	0.80	2.64	0.73	.8598	-0.06	.8098	-0.08	2.99	0.79	.2969	0.38	.2646	0.41	2.45	0.66	.4129	-0.31	.5779	-0.21
Paracentral	R	1.37	0.60	1.21	0.31	.3964	-0.30	.2761	-0.38	1.11	0.81	.2657	-0.41	.2255	-0.44	1.14	0.54	.3012	-0.39	.5043	-0.25
Pars opercularis	R	2.37	0.45	2.11	0.45	.1091	-0.57	.0484	-0.70	2.07	0.61	.1018	-0.60	.0431	-0.75	1.52	0.57	<.0001*	-1.78	.0001*	-1.64
Pars orbitalis	R	2.50	0.71	2.25	0.45	.2730	-0.39	.1728	-0.48	2.23	0.67	.2815	-0.39	.1751	-0.50	1.58	0.83	.0018	-1.25	.0035	-1.16
Pars triangularis	R	2.32	0.53	2.02	0.55	.1183	-0.55	.0384	-0.74	1.96	0.64	.0779	-0.65	.0218	-0.86	1.40	0.56	<.0001*	-1.70	.0001*	-1.61
Pericalcarine	R	0.60	0.33	0.54	0.29	.5858	-0.19	.5429	-0.21	0.48	0.36	.3476	-0.34	.3293	-0.36	0.35	0.38	.0569	-0.73	.0738	-0.69
Postcentral	R	0.82	0.47	0.85	0.37	.8281	0.08	.8927	-0.05	0.71	0.53	.5365	-0.22	.3764	-0.32	0.47	0.41	.0509	-0.75	.1403	-0.56
Posterior cingulate	R	2.75	0.33	2.62	0.54	.3487	-0.33	.3684	-0.32	2.37	0.57	.0104	-0.97	.0091	-0.98	2.41	0.28	.0073	-1.06	.0040	-1.14
Precentral	R	1.09	0.54	1.00	0.46	.5955	-0.19	.2911	-0.37	1.01	0.38	.6304	-0.17	.3771	-0.32	0.57	0.50	.0113	-1.00	.0440	-0.78
Precuneus	R	1.88	0.42	1.74	0.30	.3107	-0.36	.2525	-0.40	1.71	0.55	.3028	-0.38	.2904	-0.39	1.46	0.39	.0107	-1.00	.0190	-0.92
Rostral anterior cingulate	R	3.55	0.60	3.23	0.81	.1674	-0.49	.1883	-0.46	3.33	0.80	.3543	-0.34	.4503	-0.27	2.70	0.78	.0010	-1.33	.0010	-1.33
Rostral middle frontal	R	2.44	0.49	2.18	0.84	.2052	-0.45	.1186	-0.55	2.00	0.70	.0297	-0.81	.0165	-0.90	1.21	0.83	<.0001*	-2.16	<.0001*	-1.95
Superior frontal	R	2.47	0.59	2.23	0.60	.2357	-0.42	.1149	-0.56	2.09	0.58	.0783	-0.65	.0422	-0.75	1.47	0.85	.0002*	-1.55	.0007*	-1.38
Superior parietal	R	1.03	0.46	1.15	0.30	.4224	0.28	.4815	0.25	0.92	0.64	.5456	-0.22	.5268	-0.23	0.62	0.47	.0209	-0.90	.0352	-0.82
Superior temporal	R	2.08	0.40	1.90	0.45	.2066	-0.44	.1081	-0.57	1.87	0.54	.1780	-0.49	.0878	-0.63	1.78	0.66	.0931	-0.64	.2101	-0.48
Supramarginal	R	1.95	0.50	1.86	0.24	.5652	-0.20	.3768	-0.31	1.96	0.71	.9887	0.01	.8020	-0.09	1.45	0.48	.0095	-1.02	.0243	-0.88
Frontal pole	R	1.93	0.97	1.62	0.88	.3557	-0.32	.3276	-0.34	1.98	0.91	.8816	0.05	.8507	0.07	0.98	0.92	.0119	-0.99	.0207	-0.90
Temporal pole	R	2.15	1.03	2.31	1.36	.6941	0.14	.7121	0.13	2.14	1.02	.9856	-0.01	.9547	0.02	2.62	0.98	.2369	0.46	.1651	0.54
Transverse temporal	R	1.13	0.54	0.86	0.72	.1910	-0.46	.1362	-0.53	0.74	0.77	.0755	-0.66	.0589	-0.70	0.86	0.80	.2341	-0.45	.4079	-0.31
Insula	R	1.70	0.44	1.61	0.52	.5578	-0.20	.4129	-0.29	1.50	0.60	.2526	-0.42	.1756	-0.50	1.42	0.43	.0941	-0.64	.1785	-0.51

[1]Banks of the superior temporal sulcus

*Significant after Bonferroni correction

Table S5. Statistics for group comparison of midcortical layer MPF and mediation analysis estimating the proportion of NLI difference between HC and SSD-L mediated by midcortical MPF. Group comparison uncorrected p-value and Cohen's d (positive values indicate patients > controls) are presented. Corresponding statistics controlling age and gender are also included. R for right hemisphere; L for left hemisphere.

Region (hemisphere)		HC		SSD-L		Group comparison		Group comparison controlling age and gender		Proportion mediated[1] No cofactor	Proportion mediated[1] Controlling age and gender
		Mean	STD	Mean	STD	p-value	Cohen's d	p-value	Cohen's d	% explained	% explained
Caudal anterior cingulate	R	0.050	0.002	0.052	0.003	.0553	0.74	.0600	0.73	-	-
Lateral orbitofrontal	R	0.049	0.003	0.054	0.005	.0008*	1.36	.0023*	1.22	16%	16%
Medial orbitofrontal	R	0.049	0.003	0.056	0.006	<.0001*	1.86	<.0001*	1.79	17%	19%
Pars opercularis	R	0.051	0.002	0.052	0.003	.1959	0.49	.3032	0.39	-	-
Pars triangularis	R	0.052	0.002	0.054	0.003	.0261	0.87	.0520	0.75	-	-
Rostral middle frontal	R	0.053	0.002	0.055	0.003	.0046	1.12	.0108	1.00	-	-
Superior frontal	R	0.052	0.002	0.053	0.002	.0860	0.66	.1634	0.53	-	-
Lateral orbitofrontal	L	0.049	0.004	0.054	0.004	.0004*	1.43	.0010*	1.33	23%	20%
Medial orbitofrontal	L	0.049	0.003	0.054	0.005	.0033*	1.17	.0075	1.06	20%	19%
Pars opercularis	L	0.050	0.003	0.052	0.002	.0285	0.85	.0702	0.70	-	-
Pars orbitalis	L	0.049	0.003	0.055	0.004	.0001*	1.62	.0002*	1.55	35%	34%
Pars triangularis	L	0.051	0.003	0.055	0.003	.0003*	1.49	.0009*	1.34	-	-
Rostral anterior cingulate	L	0.047	0.002	0.051	0.004	.0013*	1.29	.0014*	1.29	-	-
Rostral middle frontal	L	0.051	0.002	0.054	0.002	.0005*	1.42	.0010*	1.33	-	-

* denotes significance after Bonferroni correction

[1] Proportion mediated data are available when pathways in the mediation model (Baron and Kenny 1986) are fulfilled (significant group effect of NLI, significant group effect of MPF, and significant association between NLI and midcortical MPF).

Table S6. Correlation statistics between NLI and illness duration for SSD in regions with significant between-group NLI differences.

Region (hemisphere)		Pearson's correlation		Controlling for age and gender	
		r	p-value	r	p-value
Pars orbitalis	L	-0.59	.0007*	-0.61	.0005*
Pars triangularis	L	-0.42	.0236	-0.38	.0483
Lateral orbitofrontal	L	-0.42	.0245	-0.35	.0650
Rostral middle frontal	L	-0.40	.0314	-0.38	.0490
Caudal anterior cingulate	R	-0.39	.0354	-0.33	.0858
Pars triangularis	R	-0.38	.0423	-0.34	.0781
Superior frontal	R	-0.37	.0502	-0.32	.0976
Rostral middle frontal	R	-0.36	.0551	-0.30	.1213
Pars opercularis	R	-0.35	.0612	-0.28	.1434
Medial orbitofrontal	R	-0.34	.0697	-0.30	.1195
Pars opercularis	L	-0.34	.0707	-0.32	.0946
Rostral anterior cingulate	L	-0.29	.1213	-0.23	.2341
Medial orbitofrontal	L	-0.27	.1495	-0.20	.3064
Lateral orbitofrontal	R	-0.27	.1599	-0.19	.3243

* denotes significance after Bonferroni correction

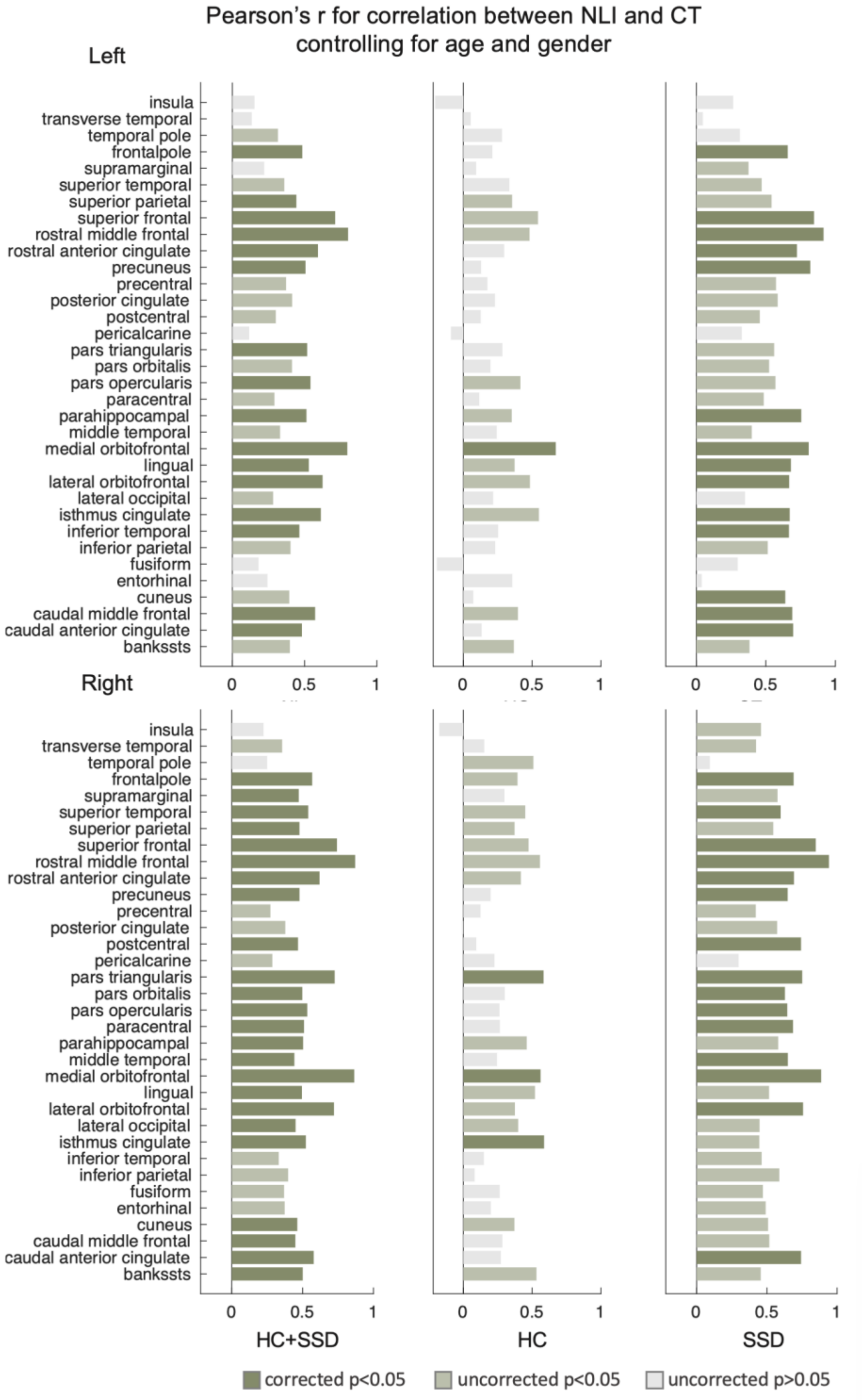

Figure S2. Associations between NLI and CT in bilateral brain areas controlling for age and gender assessed using Pearson's correlation. Significant associations are found bilaterally for a large number of regions in both the patient group (right column) and across groups (left column) with only few significant associations noted in the HC group (middle column).

Table S7. Statistics for partial correlation between NLI, CT, MPF with Symbol Span raw score controlling for age, gender, and illness duration in SSD.

Regions (hemisphere)		NLI		CT		MPF	
		Pearson's *r*	*p*-value	Pearson's *r*	*p*-value	Pearson's *r*	*p*-value
Bankssts[1]	L	0.286	.1567	0.085	.6800	0.026	.8997
Caudal anterior cingulate	L	0.103	.6176	0.233	.2511	0.134	.5136
Caudal middle frontal	L	0.658	.0003*	0.370	.0626	0.159	.4371
Cuneus	L	0.397	.0449	0.250	.2175	0.192	.3480
Entorhinal	L	0.143	.5062	0.008	.9687	0.077	.7215
Fusiform	L	-0.195	.3400	-0.005	.9816	-0.161	.4429
Inferior parietal	L	0.348	.0812	0.325	.1051	0.122	.5543
Inferior temporal	L	0.165	.4210	0.211	.3004	0.100	.6255
Isthmus cingulate	L	0.126	.5391	0.093	.6521	0.182	.3744
Lateral occipital	L	0.256	.2065	0.349	.0802	0.063	.7594
Lateral orbitofrontal	L	0.323	.1080	0.502	.0090	0.166	.4166
Lingual	L	0.032	.8773	0.119	.5641	0.100	.6285
Medial orbitofrontal	L	0.240	.2379	0.170	.4072	0.166	.4165
Middle temporal	L	0.156	.4464	0.272	.1784	0.176	.3909
Parahippocampal	L	0.353	.0765	0.107	.6044	-0.016	.9381
Paracentral	L	0.373	.0603	0.281	.1640	0.147	.4726
Pars opercularis	L	0.460	.0179	0.540	.0044	0.254	.2109
Pars orbitalis	L	0.007	.9723	0.167	.4148	0.076	.7115
Pars triangularis	L	0.398	.0439	0.550	.0036	0.205	.3162
Pericalcarine	L	0.235	.2479	0.122	.5531	0.161	.4331
Postcentral	L	0.460	.0181	0.276	.1730	0.149	.4671
Posterior cingulate	L	0.339	.0902	0.274	.1752	0.193	.3438
Precentral	L	0.502	.0090	0.537	.0047	0.113	.5840
Precuneus	L	0.437	.0254	0.375	.0592	0.165	.4214
Rostral anterior cingulate	L	0.066	.7502	0.134	.5144	0.305	.1298
Rostral middle frontal	L	0.460	.0182	0.483	.0124	0.103	.6183
Superior frontal	L	0.532	.0052	0.495	.0101	0.125	.5432
Superior parietal	L	0.561	.0028	0.414	.0357	0.200	.3278
Superior temporal	L	0.271	.1807	0.328	.1016	0.196	.3367
Supramarginal	L	0.243	.2311	0.205	.3162	0.067	.7433
Frontal pole	L	0.333	.0969	0.345	.0848	0.216	.2889
Temporal pole	L	-0.169	.4193	-0.117	.5695	0.005	.9815
Transverse temporal	L	0.071	.7306	0.375	.0588	0.304	.1306
Insula	L	0.083	.6868	0.495	.0101	0.318	.1139

Regions (hemisphere)		NLI		CT		MPF	
		Pearson's *r*	*p*-value	Pearson's *r*	*p*-value	Pearson's *r*	*p*-value
Bankssts	R	0.176	.3906	0.362	.0694	-0.014	.9465
Caudal anterior cingulate	R	0.301	.1350	0.058	.7794	0.225	.2702
Caudal middle frontal	R	0.548	.0037	0.206	.3131	0.105	.6093
Cuneus	R	0.537	.0047	0.222	.2768	0.106	.6074
Entorhinal	R	0.020	.9254	-0.073	.7236	-0.146	.5077
Fusiform	R	0.456	.0220	0.229	.2595	-0.183	.3816
Inferior parietal	R	0.253	.2116	0.180	.3782	-0.010	.9596
Inferior temporal	R	0.215	.3017	0.337	.0920	-0.187	.3696
Isthmus cingulate	R	0.044	.8316	0.297	.1402	0.120	.5592
Lateral occipital	R	0.313	.1201	0.253	.2119	-0.117	.5704
Lateral orbitofrontal	R	0.117	.5704	0.321	.1102	0.082	.6901
Lingual	R	0.082	.6923	0.216	.2883	0.032	.8772
Medial orbitofrontal	R	0.258	.2032	0.148	.4711	0.162	.4296
Middle temporal	R	0.326	.1043	0.251	.2162	0.083	.6866
Parahippocampal	R	0.241	.2349	0.242	.2344	-0.065	.7560
Paracentral	R	0.448	.0216	0.364	.0677	0.177	.3866
Pars opercularis	R	0.302	.1333	0.496	.0100	0.182	.3731
Pars orbitalis	R	0.127	.5349	0.482	.0126	0.043	.8352
Pars triangularis	R	0.429	.0289	0.384	.0530	0.227	.2654
Pericalcarine	R	0.443	.0234	0.194	.3433	0.229	.2597
Postcentral	R	0.521	.0064	0.326	.1037	0.051	.8042
Posterior cingulate	R	0.337	.0921	0.105	.6102	0.148	.4700
Precentral	R	0.508	.0081	0.333	.0966	0.051	.8042
Precuneus	R	0.451	.0208	0.124	.5450	0.116	.5714
Rostral anterior cingulate	R	0.132	.5210	0.113	.5820	0.322	.1081
Rostral middle frontal	R	0.392	.0475	0.329	.1007	0.124	.5471
Superior frontal	R	0.502	.0089	0.406	.0397	0.183	.3718
Superior parietal	R	0.418	.0337	0.073	.7232	0.130	.5259
Superior temporal	R	0.307	.1269	0.355	.0752	0.077	.7088
Supramarginal	R	0.110	.5918	0.091	.6588	0.026	.8981
Frontal pole	R	0.066	.7477	0.311	.1224	0.049	.8108
Temporal pole	R	-0.155	.4604	0.143	.4865	-0.183	.3909
Transverse temporal	R	-0.068	.7403	0.031	.8810	0.260	.1987
Insula	R	0.315	.1171	0.462	.0175	0.239	.2396

[1]Banks of the superior temporal sulcus

*Significant after Bonferroni correction

References

Manjon, J. V., Coupe, P., Buades, A., Collins, D. L., & Robles, M. (2010). MRI superresolution using self-similarity and image priors. *Int J Biomed Imaging, 2010*, 425891.

Coupe, P., Manjon, J. V., Chamberland, M., Descoteaux, M., & Hiba, B. (2013). Collaborative patch-based super-resolution for diffusion-weighted images. *Neuroimage, 83*, 245-261.

Lee, H. H., Lin, Y. C., Lemberskiy, G., Ades-aron, B., Baete, S., Boada, F. E., *et al.* (2019). SUper-REsolution TRACTography (SURE-TRACT) pipeline using self-similarity between diffusional and anatomical images. *Proc of ISMRM, Montreal, 27*.

Baron RM and Kenny DA. (1986). The moderator-mediator variable distinction in social psychological research: conceptual, strategic, and statistical considerations. *J Pers Soc Psychol, 51*(6),1173-1182.
